# Envafolimab – novel anti-PD-1/PD-L1 antibody in cancer treatment

**DOI:** 10.3389/fimmu.2026.1918429

**Published:** 2026-08-05

**Authors:** Mateusz Kciuk, Damian Kołat, Katarzyna Wanke, Renata Kontek

**Affiliations:** 1 Department of Molecular Biotechnology and Genetics, University of Lodz, Lodz, Poland; 2 Department of Functional Genomics, Faculty of Medicine, Medical University of Lodz, Lodz, Poland; 3 Doctoral School of Exact and Natural Sciences, University of Lodz, Lodz, Poland

**Keywords:** antibodies, cancer, envafolimab, immunotherapy, programmed cell death

## Abstract

The landscape of immunotherapy in oncology has markedly advanced with the development of programmed death-1 (PD-1) and programmed death ligand-1 (PD-L1) inhibitors, fundamentally reshaping cancer treatment by potentiating anti-tumor immune responses. PD-1/PD-L1 blockade agents, such as FDA-approved nivolumab, pembrolizumab, and dostarlimab, have demonstrated significant clinical efficacy across various malignancies. Additionally, investigational agents like envafolimab, a novel PD-L1 inhibitor, are currently under clinical evaluation for efficacy and safety in solid tumors. This review examines the emerging data on envafolimab and discusses strategies to optimize its therapeutic impact, including combination regimens and personalized approaches. Tailoring treatments based on individual genetic, immunological, and microbiome profiles holds the potential to enhance response rates and the durability of outcomes. The integration of these factors is pivotal in advancing the precision and success of immunotherapy in oncology.

## 1 Introduction

Cancer remains a leading cause of mortality worldwide. While the overall cancer mortality rate has steadily declined since 1991, averting more than 4 million deaths by 2021, this progress can be largely attributed to reductions in smoking prevalence, advances in early diagnostic techniques for specific cancers, and improved treatment modalities for both early- and advanced-stage cancers. However, recent trends indicate an increasing incidence in 6 of the top 10 most prevalent cancers (breast, cervical cancer, colorectal cancer, kidney, liver (female), melanoma, oropharynx, pancreas, prostate, and uterine corpus), which poses a potential threat to the gains achieved in cancer control. Between 2015 and 2019, incidence rates of breast, pancreatic, and uterine corpus cancers rose annually by 0.6% to 1%. Meanwhile, prostate, female liver, kidney, and human papillomavirus (HPV)-associated oropharyngeal cancers, along with melanoma, demonstrated higher annual increases in incidence, ranging from 2% to 3%. Notably, the incidence of cervical cancer among adults aged 30–44 years and colorectal cancer (CRC) among adults younger than 55 years has shown annual increases of approximately 1% to 2% (1).

Classical chemotherapy remains a cornerstone of cancer treatment, with its primary mechanism centered on disrupting cellular growth a hallmark of cancer cells. Chemotherapeutic agents are designed to interfere with various stages of the cell cycle, targeting rapidly dividing cells. However, due to their limited specificity for malignant cells, these agents also affect healthy proliferating cells, including those in the bone marrow, gastrointestinal tract, and hair follicles. This lack of selectivity contributes to the characteristic toxicities associated with chemotherapy, underscoring the ongoing need for more targeted and precise cancer therapies (2–4).

The advent of targeted therapies marked a significant advancement in cancer treatment. Unlike traditional chemotherapy, which non-selectively affects both malignant and healthy cells, targeted therapies are designed to interact with specific molecular targets, typically proteins or genes, that are uniquely expressed or dysregulated in cancer cells. By selectively inhibiting these cancer-specific pathways, targeted therapies can achieve more precise anti-tumor effects while minimizing collateral damage to normal tissues. This specificity not only enhances therapeutic efficacy but also reduces the incidence and severity of treatment-related side effects, representing a major step forward in the development of more tolerable cancer therapies (5).

In contrast to other therapeutic agents, small-molecule inhibitors (SMIs) are capable of permeating the cell membrane to target intracellular molecules essential for cancer cell survival and proliferation. Regorafenib is a small-molecule multi-kinase inhibitor specifically engineered to inhibit a range of kinases that play critical roles in tumor growth and maintenance. These targets include kinases involved in angiogenesis, such as vascular endothelial growth factor receptors 1–3 (VEGFR1-3) and TEK tyrosine kinase (TIE2); stromal support, including platelet-derived growth factor receptor β (PDGFR-β) and fibroblast growth factor receptor (FGFR); and oncogenic receptor tyrosine kinases, such as KIT, rearranged during transfection (RET) and rapidly accelerated fibrosarcoma (RAF). By simultaneously targeting these diverse pathways, regorafenib represents a significant advancement in cancer therapy, being the first small-molecule multi-kinase inhibitor to demonstrate a meaningful survival benefit in patients with metastatic cancer who have exhausted standard treatment options. This multi-target approach has shown promise in overcoming resistance and improving outcomes in challenging patient populations (6).

A third approach in cancer treatment is immunotherapy, which shifts the focus from directly targeting tumor cells to modulating the patient’s immune system. The primary goal of immunotherapy is to stimulate and enhance the immune system’s natural ability to recognize, target, and eradicate cancer cells. By mobilizing immune components, such as T cells, natural killer cells (NKs), and antibodies, immunotherapy seeks to initiate a robust and sustained immunological response against the tumor. This approach aims to overcome immune evasion mechanisms employed by tumors and to promote long-term immunological memory, potentially preventing recurrence. In recent years, cancer immunotherapy has gained increasing prominence as a treatment strategy, often surpassing traditional therapies in both research attention and clinical innovation due to its potential for durable and personalized responses (7, 8).

Immunotherapy offers a promising treatment approach for some patients, particularly those with high microsatellite instability (MSI-H) or mismatch repair deficiency (dMMR) tumors. It can lead to durable responses and has the potential to achieve complete remission. Immunotherapeutic strategies include immune checkpoint inhibitors (ICIs), cancer vaccines, adoptive cell therapies (e.g., chimeric antigen receptor T-cell (CAR-T) therapy and tumor-infiltrating lymphocyte (TIL) therapy), and cytokine-based therapies. Among these approaches, ICIs are the most extensively studied and clinically established in this setting (9, 10). ICIs, such as pembrolizumab (Keytruda) and nivolumab (Opdivo), have received Food and Drug Administration (FDA) approval for the treatment of advanced cancers and represent the best-studied immunotherapeutic agents used in combating this disease. Additionally, the FDA issued permission on August 17, 2023, for the use of dostarlimab (Jemperli) in adult individuals diagnosed with recurrent or advanced solid tumors with dMMR (11–14).

**Figure 1:**
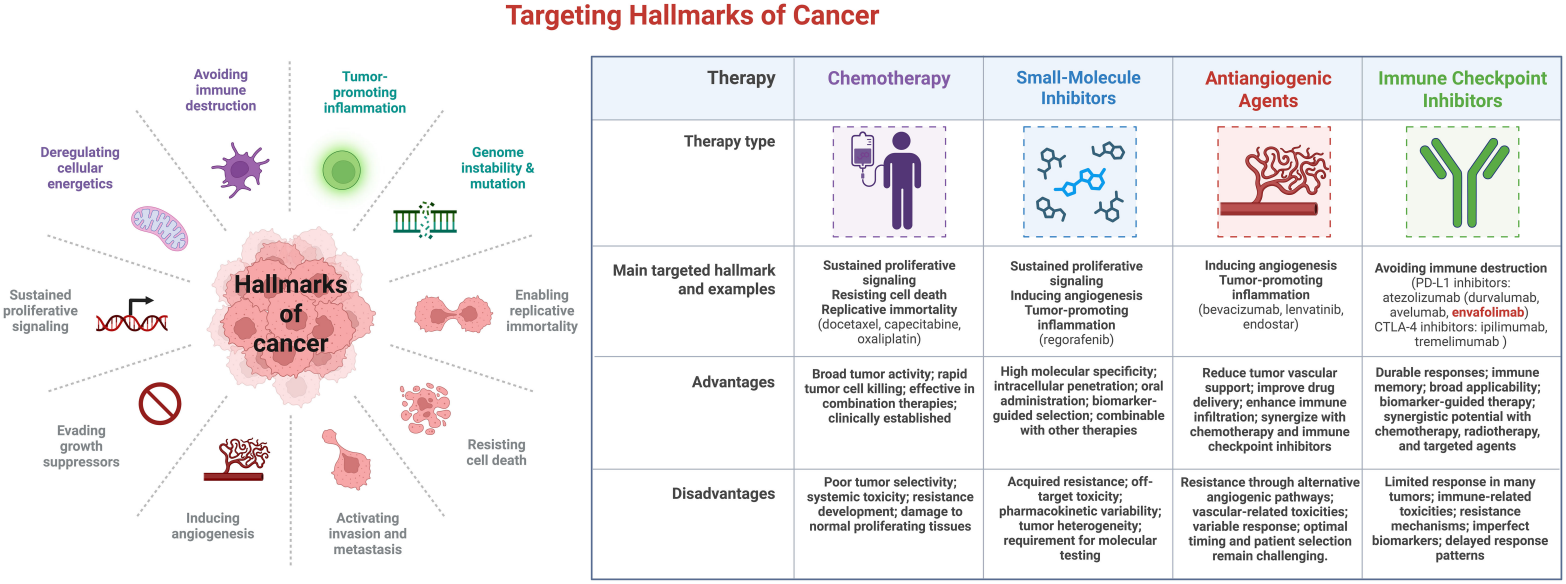
Overview of major therapeutic strategies targeting the hallmarks of cancer: mechanisms, advantages, and limitations. Schematic representation of the major therapeutic approaches used to target cancer hallmarks, including classical chemotherapy, small-molecule inhibitors, antiangiogenic agents, and immune checkpoint inhibitors. The figure summarizes the primary mechanisms of action, therapeutic advantages, and key limitations of each strategy. Created in BioRender. Kciuk, M. (2026) https://BioRender.com/cdewhqf.

**Figure 1** presents the concept of hallmarks of cancer and a short overview of major cancer treatment approaches, including classical chemotherapy, use of small-molecule inhibitors (targeted therapy), antiangiogenic agents that are commonly combined with immune checkpoint inhibitors (including PD-1/PD-L1 inhibitors) for cancer treatment (**Figure 1**).

Envafolimab is an innovative monoclonal antibody employed in cancer immunotherapy, specifically designed to target and inhibit programmed death-ligand 1 (PD-L1). PD-L1 plays a critical role in immune evasion by binding to the programmed death 1 (PD-1) receptor on T cells, thereby suppressing the anti-tumor immune response. By binding to PD-L1, envafolimab blocks this interaction, enhancing T-cell activation and promoting immune-mediated tumor cell destruction. Envafolimab is under investigation in multiple clinical trials to evaluate its efficacy and safety across a range of malignancies, including advanced solid tumors. One distinguishing feature of envafolimab is its subcutaneous route of administration, which offers a more convenient alternative to the intravenous administration required for many other PD-L1 inhibitors, potentially improving patient compliance and quality of life.

Despite the rapid advancement of immune checkpoint blockade, a comprehensive assessment of envafolimab that integrates its unique molecular characteristics, clinical development, and potential role within the evolving immuno-oncology landscape remains limited. This review addresses this knowledge gap by providing a focused analysis of the mechanistic basis, preclinical evidence, clinical outcomes, and future opportunities for optimizing envafolimab-based therapeutic strategies. Given the increasing need for effective, accessible, and patient-centered immunotherapies, understanding the distinctive features of envafolimab, particularly its subcutaneous administration and expanding clinical applications, is of particular importance for guiding future research and clinical decision-making.

## 2 PD-1/PD-L1 checkpoint and antitumor immune response

Tumor cells are identified by the adaptive immune system, which consists of B and T cells, as well as the innate immune system, mostly through natural killer (NK) cells. Effector CD4+ and cytotoxic CD8+ T lymphocytes can identify peptide antigens displayed on major histocompatibility complex (MHC) class II or MHC class I, respectively (15, 16).

**Figure 2:**
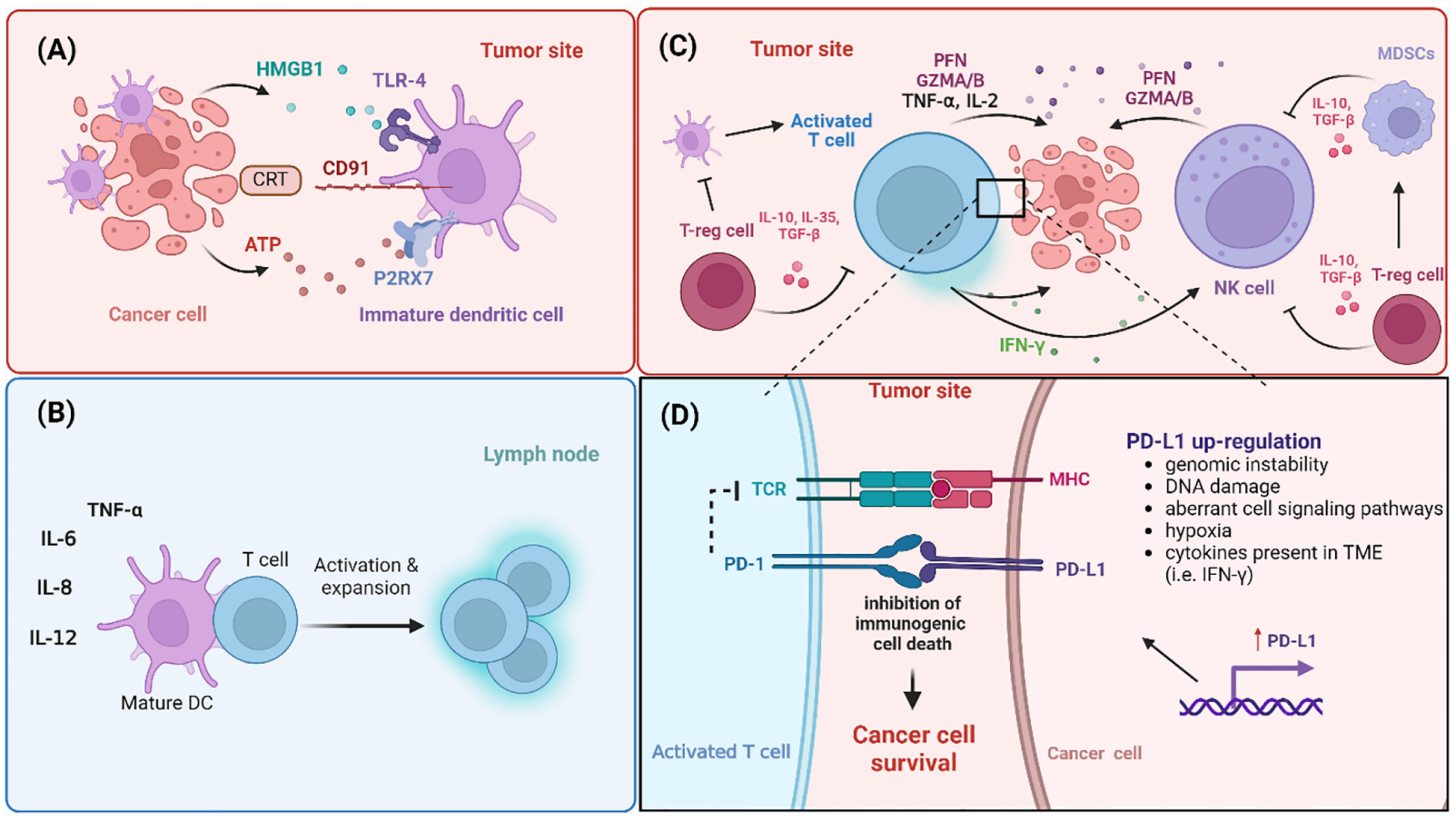
Overview of anti-cancer immune response. **(A)** Release of DAMPs, including HMGB1, CRT, and ATP, from dying cancer cells and their recognition by DCs. **(B)** Antigen uptake and presentation by DCs leading to activation of adaptive antitumor immunity. **(C)** Cytotoxic activity of activated CD8⁺ T lymphocytes mediated by perforins, granzymes, IFN-γ, and TNF-α. **(D)** Regulation of PD-L1 expression by genomic instability, oncogenic signaling, and epigenetic mechanisms, promoting immune escape and an immunosuppressive tumor microenvironment. A detailed description of the figure is provided in the main text. Full names of all abbreviations are listedin the glossary section. Created in BioRender. Kciuk, M. (2025) https://BioRender.com/ec8skco.

Immunogenic cell death (ICD) is linked to the liberation of damage-associated molecular patterns (DAMPs) from dying cells. DAMPs whether present on cell surfaces or secreted extracellularly, can enhance tumor antigen presentation and promote adaptive immunity. Cancer cells release high-mobility group box 1 protein (HMGB1), which functions as a ligand for the toll-like receptor 4 (TLR-4) on dendritic cells (DCs). Additionally, the calreticulin (CRT) expressed by cancer cells interacts with the CD91 receptor on DCs. In contrast, adenosine triphosphate (ATP) produced from cancer cells interacts with the P2X purinoceptor 7 (P2RX7) on DCs (17) (**Figure 2A**).

DCs are necessary for the initiation of cytotoxic CD8+ T cell activation. During this process, DCs capture antigens from tumor cells that are undergoing apoptosis, and with the assistance of CD4+ helper cells, they present these antigens to CD8+ T lymphocytes. In addition, DCs can release tumor necrosis factor α (TNF-α) as well as interleukins (IL-6, IL-8, and IL-12), which have a role in initiating the immune response to cancer (18) (**Figure 2B**).

Activated CD8+ T lymphocytes can eliminate cancer cells by releasing granzyme A/B (GZMA/B), perforins (PFNs), interferon-gamma (INFγ), and TNFα (19–21) (**Figure 2C**), which can be impeded by the over-production of programmed death ligand-1 (PD-L1) by cancer cells as a result of inherent genomic instability, aberrant cell signaling or in response to changes in tumor microenvironment (TME) such as hypoxia or the presence of certain cytokines (**Figure 2D**), and lead to formation of immunosuppressive TME rich in regulatory T cells (T-regs) and myeloid-derived suppressor cells (MDSCs), which inhibit the function of activated T cells and NK-cells (22, 23).

The PD-1/PD-L1 checkpoint is therefore an essential regulatory mechanism of the immune system, which has a pivotal function in preserving immunological balance and preventing autoimmunity. Nevertheless, cancer cells frequently exploit this pathway to evade immune surveillance, resulting in tumor development and progression (24).

PD-1 (CD279) is expressed on the surface of activated T cells, B cells, and NK cells. The primary function of PD-1 is to act as a negative regulator of immune responses, providing a “brake” to prevent over-activation of the immune system. PD-1 inhibits T cell proliferation, cytokine generation, and cytotoxic activity of immune cells via interaction with the ligands (PD-L1 and PD-L2) present on the surface of other cells. This inhibition is crucial for maintaining immune tolerance and preventing autoimmunity, but it can be detrimental when exploited by tumor cells (8, 25).

The transmembrane glycoprotein PD-L1, also referred to as CD274, is primarily expressed on tumor cells and, to a lesser extent, on various non-tumor cells within the TME, including macrophages, DCs, and certain stromal cells. As previously mentioned, inflammatory signals within the TME, such as IFN-γ, can induce the expression of PD-L1 in cancer cells. IFN-γ is produced by activated T cells and other immune cells in response to tumor antigens. The up-regulation of PD-L1 in response to IFN-γ represents a feedback mechanism that dampens the immune response and promotes immune tolerance within the tumor (26, 27). The TME, which is defined by characteristics such as low oxygen levels, lack of nutrients, and interactions with stromal cells, can affect the expression of PD-L1 in cancer cells. Hypoxia-inducible factors (HIFs), which become active in low oxygen conditions within the tumor can directly increase the expression of PD-L1 (27–31). Moreover, bidirectional crosstalk between tumor cells and components of the TME, including cancer-associated fibroblasts (CAFs) and infiltrating immune cells, regulates *PD-L1* expression through paracrine signaling mediated by cytokines and growth factors (32–34).

Genomic instability, considered the hallmark of cancer, results from particular genetic changes, such as gene amplifications, chromosomal rearrangements, or specific mutations, that might cause increased PD-L1 expression (35–38). This can lead to the abnormal activation of signaling pathways in cancer cells but can also stimulate the production of PD-L1. For instance, mutations in genes like epidermal growth factor receptor (*EGFR*) (39, 40) and Kirsten rat sarcoma viral oncogene homolog (*KRAS)* (41, 42) which are prevalent in different types of malignancies, might result in elevated PD-L1 expression.

Alterations in DNA methylation and histone modifications may control the expression of PD-L1. Reduced methylation in the promoter region of the *PD-L1* gene can result in increased production of this protein in cancer cells. Epigenetic regulation can be affected by different causes, including environmental stresses and oncogenic signaling (38, 43–45) (**Figure 2D**).

PD-L2 (CD273) is also a transmembrane glycoprotein, primarily found on antigen-presenting cells (APCs), including DCs and macrophages. While PD-L2’s role in immune regulation is similar to PD-L1, its expression in tumor cells is less common and its contribution to cancer immune evasion is less understood (8, 25).

In summary, the PD-1/PD-L1 pathway is a crucial mechanism via which tumor cells avoid being detected by the immune system. PD-1/PD-L1 interactions in the TME can lead to various immune-suppressive outcomes, including:

- T cell exhaustion: Chronic engagement of PD-1 by PD-L1 leads to a state of functional impairment of T cells. T-cell exhaustion arises when T cells are consistently activated, commonly seen in cases of chronic infections and malignancies. T cells in this condition experience a progressive decline in their capacity to adequately react to antigens, leading to diminished immune responses against infections or malignancies. Exhausted T cells exhibit reduced synthesis of effector cytokines, such as IFN-γ, TNF-α, and interleukin-2 (IL-2). Exhausted T lymphocytes exhibit impaired proliferation and reduced effector functions in response to persistent antigenic stimulation, limiting their ability to eliminate tumor cells. Consequently, diminished antitumor immune surveillance facilitates tumor progression, including sustained tumor cell proliferation, local invasion, and metastatic dissemination (46–49).
- T-reg expansion: The PD-1/PD-L1 pathway can promote the expansion of T-reg cells which suppress the activation of effector T cells. This can be accomplished either by direct contact between cells or via the release of immunosuppressive cytokines including IL-10 (inhibits the production of pro-inflammatory cytokines and dampens the activity of DCs, reducing the activation of effector T cells), transforming growth factor-beta (TGF-β) (inhibits the growth and stimulation of T cells and can also enhance the development of new T-reg cells) and IL-35 (cytokine, which is also produced by T-reg cells, exhibits strong immunosuppressive properties by diminishing the proliferation and functionality of T cells). T-reg cells can trigger apoptosis in effector T cells, hence further reducing the anti-tumor immune response. Furthermore, they can mobilize more T-reg cells and engage with other immunosuppressive cells, such as MDSCs, and suppress the function of APCs, such as DCs (50–54).
- Suppression of tumor-associated macrophages (TAMs) and NK cells: PD-L1 expression on tumor cells can inhibit TAM and NK cell activity, reducing their capacity to mount effective anti-tumor responses (25, 55, 56). TAMs are an essential component of the TME, capable of either facilitating or impeding tumor growth according to their polarization state. TAMs are commonly classified into two main phenotypes:

M1 macrophages have pro-inflammatory and anti-tumor properties by releasing cytokines that stimulate immune responses and directly eliminate tumor cells.

M2 macrophages have anti-inflammatory properties and promote tumor growth by facilitating tissue repair, angiogenesis, and immune evasion. PD-L1, which is produced by tumor cells and other elements of the TME, can attach to PD-1 receptors on macrophages. This interaction enhances M2 polarization, resulting in decreased anti-tumor activity and heightened immunosuppressive effects. The interaction between PD-L1 and PD-1 can hinder the activation of macrophages, leading to a decrease in the production of pro-inflammatory cytokines such as TNF-α and IL-12. This diminishes the capacity of TAMs to provoke anti-tumor immune responses (57–61). NK cells play a vital role in the innate immune system by identifying and eliminating cancer cells by different methods, such as releasing cytotoxic granules and producing IFN-γ. NK cells can express PD-1 on their surfaces, and when they come into contact with PD-L1 on tumor cells or other cells in the TME, it can hinder their activation (62, 63).

The therapeutic blockade of the PD-1/PD-L1 pathway has emerged as a powerful strategy for reactivating the immune system against cancer. mAbs that specifically bind to PD-1 or PD-L1 can interfere with this immunological checkpoint, hence restoring the normal functioning of T cells and facilitating a strong immune response against cancer cells. This has led to improved clinical outcomes, including increased progression-free survival (PFS) and overall survival (OS) in various cancer types (25, 64).

## 3 Envafolimab

Heavy-chain antibodies (HCAbs) represent a remarkable deviation from the conventional antibody structure, composed solely of heavy chains and lacking the light chains found in typical immunoglobulins. Discovered around thirty years ago in camelids, including species such as camels, llamas, dromedaries, and vicuñas, these antibodies have since emerged as valuable assets in both scientific and clinical fields. HCAbs offer numerous advantages that make them especially appealing for biomedical applications. Their compact structure grants them superior tissue penetration, while their high thermal and chemical stability allows them to remain functional under challenging conditions. Furthermore, their ability to bind targets with high specificity and affinity, combined with a lower risk of provoking immune responses in humans, enhances their utility in therapeutic and diagnostic contexts. Another significant benefit is their ease of production, which makes them cost-effective and amenable to large-scale manufacturing. Additionally, HCAbs can be engineered into multivalent or multi-specific constructs, further amplifying their versatility (65).

Envafolimab (KN035) is a first-in-class subcutaneous PD-L1 inhibitor derived from a humanized single-domain antibody (sdAb) fused to a human IgG1 Fc fragment, combining the compact size and structural flexibility of nanobodies with the effector properties of conventional antibodies (66, 67). Unlike classical monoclonal antibodies such as pembrolizumab and nivolumab, which contain paired heavy- and light-chain variable domains, envafolimab consists of a camelid-derived heavy chain-only variable domain that retains high antigen specificity while exhibiting improved tissue penetration and stability. Structural characterization of the envafolimab–PD-L1 complex demonstrated that the antibody-binding domain targets a conserved, relatively flat surface of PD-L1 that overlaps with the PD-1 binding interface, thereby competitively disrupting PD-1/PD-L1 signaling. The interaction is primarily mediated by a 21-amino-acid complementarity-determining region 3 (CDR3) loop that forms short helical structures and establishes critical hydrophobic and ionic contacts with PD-L1 residues, including Ile54, Tyr56, and Arg113. Mutational analyses identified key PD-L1 hotspot residues required for binding and revealed that envafolimab binds PD-L1 with approximately 1000-fold greater affinity than the endogenous PD-1 receptor, while exhibiting no detectable binding to PD-L2, providing a structural explanation for its selectivity and potential reduction of off-target immune modulation (68).

Biophysical and functional studies further demonstrated that envafolimab binds wild-type human PD-L1 with high affinity (dissociation constant of approximately 3.0 nM) and blocks PD-L1 interaction with PD-1 with an IC_50_ of approximately 5.25 nM, without cross-reactivity toward *PD-L1* mutants or murine PD-L1. *In vitro* mixed lymphocyte reaction assays showed that envafolimab dose- and time-dependently enhanced T-cell activation and cytokine production, including IFN-γ release from CD4+ T cells, with activity comparable to or exceeding that of durvalumab at equivalent molar concentrations. Furthermore, in a humanized immune xenograft model consisting of *PD-L1*-overexpressing A375 tumor cells and human peripheral blood mononuclear cells (PBMCs), envafolimab significantly inhibited tumor growth, demonstrating anti-tumor efficacy similar to or greater than that of durvalumab (66, 69).

Later experiments demonstrated that envafolimab effectively inhibited the proliferation, invasion, and migration of gastric cancer cells with low *PD-L1* expression, while also inducing apoptosis. The DEAD-box helicase 20 (DDX20) protein was identified as a potential secondary target of envafolimab in these cells, with its role linked to the nuclear factor NF-kappa-B (NF-κB) signaling pathway. Western blot analysis revealed a reduction in the protein levels of DDX20, NF-κB p65, and TNF-α following treatment with envafolimab. Additionally, silencing the *DDX20* gene using small interfering RNA (siRNA) further elucidated its role in the effects observed in gastric cancer cells with low *PD-L1* expression. These findings suggest new potential mechanisms of action for envafolimab, independent of its role as a PD-L1-targeting agent (70).

Envafolimab was designed for subcutaneous administration, making it more convenient compared to traditional intravenous infusions. The complete therapeutic dosage of envafolimab, which is 150 mg, can be administered as a single subcutaneous injection of 0.75 ml in less than 30 seconds. This is possible because the fusion protein has a high solubility of 200 mg/mL. The subcutaneous route can be advantageous for patient comfort and compliance. The simplicity of subcutaneous injections might allow for home administration in the future, increasing patient convenience (66, 67).

From a mechanistic standpoint, SC-administered monoclonal antibodies, including envafolimab, are predominantly absorbed via the lymphatic system, with studies showing that approximately 63–72% of the administered dose is transported through lymphatics, contributing to the majority of systemic bioavailability. This lymphatic uptake results in markedly higher drug exposure within draining lymph nodes, up to an order of magnitude greater than systemic circulation. Such a distribution pattern is particularly advantageous in immuno-oncology, as lymph nodes are critical sites for antigen presentation and T-cell activation, suggesting that envafolimab may more effectively engage and modulate immune responses at their initiation points. Additionally, lymphatic-directed delivery may promote more sustained and physiologically relevant immune activation, potentially enhancing antitumor efficacy while avoiding the high peak plasma concentrations sometimes associated with IV dosing. Coupled with its favorable pharmacokinetic profile, this targeted exposure could also contribute to a manageable safety profile (71).

In November 2021, envafolimab achieved two significant milestones: it became the first PD-L1 inhibitor developed in China to receive approval, and it also became the first subcutaneously injected PD-L1 inhibitor to be approved globally (72).

## 4 Clinical data on envafolimab

### 4.1 Safety and pharmacokinetics

In the first open-label phase I trial (NCT02827968), twenty-eight patients with previously treated advanced solid tumors received subcutaneous envafolimab, with dosing in two phases: a dose-escalation phase where doses ranged from 0.01 to 10 mg/kg weekly, and a dose-exploration phase where ten patients received 300 mg every four weeks. No dose-limiting toxicities (DLTs) or injection-site adverse reactions were observed. Common ICI-associated adverse effects, including fatigue, nausea, diarrhea, and hypothyroidism, were reported. Envafolimab demonstrated a favorable pharmacokinetic profile across the 0.01–10 mg/kg dosing range, with both the area under the concentration–time curve (AUC) and maximum plasma concentration (C_max) increasing proportionally with dose. Peak plasma concentrations were typically achieved within four to seven days post-administration. In the dose-exploration phase, the terminal half-life was approximately 14 days after the initial dose in the first treatment cycle, extending to 23 days at steady-state conditions. Notably, three patients achieved a confirmed partial response (PR), underscoring envafolimab’s potential efficacy in this cohort (73).

An open-label, multicenter phase I trial (NCT03248843) assessed the safety, tolerability, pharmacokinetics (PK), and efficacy of envafolimab as monotherapy in Japanese patients with advanced solid tumors. In the dose-escalation phase, ten patients received weekly subcutaneous envafolimab at doses of 1.0 mg/kg, 2.5 mg/kg, and 5.0 mg/kg. In the subsequent dose-expansion phase, sixteen patients were administered 2.5 or 5.0 mg/kg biweekly, and an additional nine patients received 300 mg every four weeks. No DLTs were reported, indicating a favorable tolerability profile. Envafolimab’s safety profile was consistent with those of similar PD-L1 inhibitors, with no novel adverse events observed. Three patients experienced grade ≥ 3 adverse events potentially related to envafolimab, including adrenal insufficiency, cerebral infarction, and immune-mediated enterocolitis. Pharmacokinetic analysis revealed a proportional increase in both the AUC and C_max with increasing doses. Efficacy outcomes demonstrated an overall response rate (ORR) of 11.4% and a disease control rate (DCR) of 34.3%, suggesting potential clinical benefit in this patient population (74).

In a retrospective, multicenter cohort study including 58 patients with advanced disease, envafolimab demonstrated a manageable safety profile, with treatment-emergent adverse events (TEAEs) observed in approximately half of the patients and immune-related adverse events (irAEs) in around one-quarter, without reports of grade 5 toxicity. The most common toxicities included dermatologic and pulmonary events, the latter appearing at a relatively higher frequency compared to previous studies, suggesting potential tumor-type-specific susceptibility. Importantly, no clear clinical predictors of irAE occurrence were identified, and a substantial proportion of patients with prior immunotherapy-related toxicity did not experience recurrence, indicating a potential role for envafolimab in rechallenge strategies. From an efficacy perspective, envafolimab achieved a median OS of 8.5 months and progression-free survival of 6.1 months, with an objective response rate of approximately 19% and DCR close to 70%. These outcomes are broadly consistent with those reported for other ICIs, such as pembrolizumab or nivolumab, particularly when accounting for the inclusion of patients with poorer performance status and later lines of therapy in real-world settings. Notably, subgroup analyses identified Eastern Cooperative Oncology Group performance status, advanced tumor stage, and later-line treatment as significant predictors of poorer prognosis, underscoring the importance of patient selection in optimizing therapeutic benefit. Furthermore, exploratory analyses suggested a potential association between PD-L1 expression and improved progression-free survival, although statistical significance was not reached, and a possible link between EGFR mutation status and irAE incidence, which requires further validation (75).

Based on data from four clinical studies (NCT02827968, NCT03101488, NCT03248843, and NCT03667170), a population pharmacokinetic modeling approach was conducted to select an appropriate dosing regimen for envafolimab in subsequent studies. Simulations were performed to compare three different dosing regimens: a weight-based dose (2.5 mg/kg weekly), a fixed dose of 150 mg weekly, and a fixed dose of 300 mg every two weeks. The aim was to determine whether a simplified fixed dosing schedule could be as effective as the weight-based regimen. The analysis found no significant difference in efficacy between the weight-based and fixed-dose regimens, indicating that both approaches were equally effective. These results support the use of either a 150 mg weekly or 300 mg biweekly fixed dosing regimen for treating patients with solid tumors, as both regimens offer a favorable balance between effectiveness and safety (76).

### 4.2 Envafolimab in dMMR/MSI-H tumors

In 2017, results from a phase 2 study indicated that nivolumab administration led to long-lasting positive outcomes and effective management of disease progression in individuals with metastatic CRC characterized by dMMR or MSI-H who had previously been treated with fluoropyrimidine and oxaliplatin or irinotecan (77). The mismatch repair (MMR) is a DNA repair process that plays a crucial role in the identification and correction of errors or mismatches that arise during the replication of DNA molecules. In the context of CRC, certain individuals may exhibit a defect or impairment in the MMR system. Deficiency in the MMR pathway can arise from two mechanisms: (a) point mutations can occur in one of the several genes that encode a protein involved in the MMR repair pathway, or (b) the suppression of promoter regions of MMR genes through hypermethylation in an epigenetic manner. Regardless of the mechanism, deficiency in functional MMR proteins compromises their ability to accurately identify and rectify errors that arise during DNA replication. The insufficient functioning of the MMR machinery leads to the buildup of genetic alterations within the DNA. Mutations have the potential to manifest across the entire genome, however, they exhibit a greater degree of prominence within regions containing microsatellites. Microsatellites are genomic regions characterized by tandemly repeated short DNA sequences. MSI-H is a pathological state distinguished by the abundance of mutations observed in microsatellite DNA sequences within a tumor (>12 mutations per 106 DNA bases) (78–82). While dMMR/MSI-H status is more common in CRC (5-15%) (83), endometrial (20-30%) (84), and ovarian cancers (20%) (85), it is not confined to these types. Non-small cell lung cancer (NSCLC), breast cancer, brain cancer, prostate cancer, pancreatic cancer, bladder cancer, melanoma, and several other cancers may also exhibit dMMR/MSI-H, albeit at lower frequencies (86–88).

dMMR/MSI-H tumors have a high mutational load referred to as tumor mutational burden (TMB), which results in the production of many abnormal proteins recognized by the immune system. TMB refers to the number of nonsynonymous mutations per coding area present in the DNA of a tumor. It is a biomarker that can be used to assess the genetic complexity of a tumor and its potential response to certain therapies. TMB has been shown to strongly correlate with objective responses to ICIs, making it a valuable predictive biomarker. The presence of TMB is linked to the generation of atypical proteins that undergo proteolytic degradation, resulting in the formation of neoantigens. These neoantigens are subsequently identified by T-cells, which trigger the release of interferons (IFNs) and subsequently stimulate the up-regulation of interferon regulatory factor (IRF) and PD-L1 to which targeted agents can bind (25, 81, 89, 90). Elevated levels of clonal neoantigens are associated with a positive response, whereas subclonal neoantigens, often resulting from tumor heterogeneity, may be indicative of resistance to immunotherapy (91–93).

Moreover, some studies suggest that tumors with homologous recombination deficiency (HRD) may exhibit increased sensitivity to immunotherapy. HRD tumors have a higher degree of immunogenicity, rendering them more responsive to therapeutic intervention through the use of ICIs. The co-administration of ICIs and poly(ADP-ribose)polymerase (PARP) inhibitors has been proposed as a potential therapeutic approach for cancers with HRD (94, 95). In addition, both cells with base excision repair (BER) and MMR deficiencies exhibit an increased production of neoantigens, resulting in the up-regulation of PD-L1. Furthermore, it is possible that cells lacking functional homologous recombination (HR) and non-homologous end joining (NHEJ) pathways may exhibit enhanced responsiveness to anti-PD-L1 therapies. This heightened sensitivity could be attributed to the release of DAMPs triggered by prior or concurrent chemotherapy or radiotherapy (25, 96).

A single-arm, phase 2 trial (NCT03667170) was conducted to evaluate the efficacy and safety of envafolimab in patients with advanced dMMR/MSI-H tumors who had previously undergone treatment. Patients with histologically confirmed locally advanced or metastatic dMMR/MSI-H solid tumors were enrolled, including 65 with CRC, 18 with gastric cancer, and 12 with other solid tumors. Participants received weekly subcutaneous injections of envafolimab at a dose of 150 mg for a 28-day treatment cycle. Efficacy endpoints included an ORR of 42.7%, a DCR of 66% (defined as complete/PR or stable disease), duration of response (DoR) of 92.2% of patients maintaining response for >12 months, PFS of 11.1 months, and OS not reached at the time of data reporting. TEAEs were reported in 96% of patients, with 37% experiencing at least one grade 3 or 4 TEAE. Three grade 5 TEAEs occurred, including lower gastrointestinal perforation, cholestatic jaundice, and death. However, these events were not deemed to be related to envafolimab treatment (67).

A separate study was conducted to evaluate the safety and efficacy of neoadjuvant subcutaneous envafolimab in patients with locally advanced colon cancer characterized by dMMR/MSI-H.

A total of 15 patients were enrolled. Following treatment, CR was observed in six patients, PR in five, and SD in four. Among the patients who experienced a clinical CR, three chose a “watch and wait” approach, while surgery was performed on twelve patients. Postoperative pathology revealed that seven patients had achieved a pathological CR (pCR), and five demonstrated tumor regression, resulting in an overall CR rate of 66.7%. The most common TEAEs were pruritus and rash, occurring in 40% of cases, with no instances of severe adverse events. No recurrence of disease was observed during the 7.9-month follow-up period. These findings suggest that envafolimab demonstrated favorable efficacy and safety in patients with locally advanced colon cancer characterized by dMMR/MSI-H (97, 98).

In a recent open-label, single-arm phase II study, Fang et al. investigated the neoadjuvant use of envafolimab in patients with dMMR locally advanced colorectal cancer, a molecular subtype known for high responsiveness to immune checkpoint blockade but limited benefit from conventional chemotherapy. A total of 15 patients were enrolled, with 13 evaluable for efficacy after receiving three to four cycles of subcutaneous envafolimab (300 mg every three weeks). The study demonstrated a notable complete response (CR) rate of 53.8%, including both clinical and pCR, with a relatively rapid median time to response of 3.1 months. Among the treated cohort, 11 patients proceeded to radical surgery, achieving R0 resection without observed recurrence in the follow-up period, underscoring the potential of neoadjuvant PD-L1 blockade to induce profound tumor regression in dMMR disease. Importantly, the safety profile was favorable, with treatment-related adverse events reported in only 28.6% of patients and limited to predominantly low-grade toxicities, with a single grade 3 event. Beyond efficacy, the subcutaneous route of administration contributed to high patient convenience and satisfaction, highlighting a practical advantage over intravenously delivered checkpoint inhibitors. Collectively, these findings suggest that envafolimab represents a promising and well-tolerated neoadjuvant immunotherapeutic strategy in dMMR colorectal cancer, although validation in larger, controlled trials is required to establish its comparative effectiveness against established PD-1–based regimens (99).

### 4.3 Envafolimab combined with chemotherapy and radiotherapy

The PRECAM trial was conducted to evaluate the potential synergy between short-course conventional neoadjuvant capecitabine and oxaliplatin (CAPEOX)-based chemotherapy, radiotherapy, and envafolimab in locally advanced rectal cancer (LARC). Preliminary results indicated a promising pCR rate of 62.5% and pathologic response rate of 75%. The treatment regimen demonstrated an acceptable safety profile, with the most common treatment-related adverse effects (TRAEs) being tenesmus, diarrhea, and leukopenia (100).

In a retrospective, single-arm study of 36 patients with LARC, Fan et al. evaluated the integration of the PD-L1 inhibitor envafolimab with standard neoadjuvant chemoradiotherapy (nCRT), providing important insights into both clinical efficacy and TME modulation. Patients with stage T3+/N1–2/M0 disease received long-course radiotherapy with concurrent capecitabine, followed by XELOX chemotherapy plus envafolimab prior to surgery. Histopathological and immunohistochemical analyses were performed on paired tumor samples, with TILs, including CD4+ T cells, CD8+ T cells, and CD56+ natural killer (NK) cells, quantified and graded based on density. The study reported a notably high pCR rate of 47.2%, alongside substantial tumor downstaging (94.4% for T stage and 86.1% for N stage). Importantly, treatment was associated with a significant increase in immune cell infiltration, particularly CD8+ T cells, suggesting enhanced cytotoxic immune activation within the TME. While higher baseline TIL densities were associated with improved tumor regression grades (TRG 0–2), these correlations did not reach statistical significance, likely reflecting the limited sample size. Methodologically, the study employed standardized immunohistochemistry protocols and rigorous pathological assessment, although it lacked prospective design, power calculations, and stratification by MSI/MMR status. Overall, these findings support the hypothesis that combining nCRT with PD-L1 blockade can potentiate antitumor immunity in LARC, while also highlighting the need for larger, biomarker-driven studies to validate these observations and refine patient selection (101).

In a prospective, single-arm phase II trial conducted at the Fifth Medical Center of the Chinese PLA General Hospital, Zhao et al. evaluated the clinical efficacy, safety, and biomarker landscape of envafolimab combined with carboplatin–etoposide as first-line therapy in patients with extensive-stage small-cell lung cancer (ES-SCLC). The study enrolled 32 treatment-naïve patients meeting strict eligibility criteria, including ECOG performance status 0–1 and measurable disease per RECIST 1.1, who received four induction cycles of chemotherapy plus subcutaneous envafolimab followed by maintenance immunotherapy until progression or intolerance. With a median follow-up of 27.7 months, the regimen demonstrated robust antitumor activity, yielding an objective response rate of 87.1% and DCR of 100%, alongside a median progression-free survival of 6.43 months and OS of 20 months, outcomes that compare favorably with historical data from chemo-immunotherapy trials in ES-SCLC. Importantly, the safety profile was manageable, with TRAEs observed in 59.4% of patients and grade ≥3 events in only 15.6%, and no treatment-related deaths reported, supporting the tolerability of this subcutaneously administered PD-L1 inhibitor. Beyond clinical efficacy, exploratory serum proteomic profiling using proximity extension assay technology identified several immune-related proteins, namely C-C motif chemokine ligand 3 (CCL3), C-X-C motif chemokine ligand 10 (CXCL10), hepatocyte growth factor (HGF), and C-X-C motif chemokine ligand 8 (CXCL8), as potential prognostic biomarkers, with elevated levels correlating with inferior survival outcomes. These findings suggest that systemic immune signatures reflecting an immunosuppressive TME may influence therapeutic resistance and disease progression. Collectively, this study highlights the promise of envafolimab-based chemo-immunotherapy as an effective and convenient first-line strategy in ES-SCLC, while underscoring the need for larger, randomized trials and biomarker-driven patient stratification to refine treatment selection (102).

Recent clinical investigations have explored the safety and efficacy of envafolimab-based combinations across various hepatocellular carcinoma (HCC) settings. In a multicenter, single-arm phase II study of first-line therapy for unresectable HCC, treatment with weekly subcutaneous envafolimab (150 mg) plus weight-based lenvatinib (12 mg for patients weighing over 60 kg, 8 mg for patients weighing under 60 kg; an anti-angiogenic drug) yielded a median OS of 18.5 months and a median PFS of 9.4 months. Among 30 evaluable patients, the ORR and DCR were 40% and 80%, respectively, with grade ≥ 3 TRAEs in 30% of patients, and no treatment-related deaths (103). Building on these results, a cohort study integrating transcatheter arterial chemoembolization (TACE) followed by envafolimab and lenvatinib in 36 patients reported an ORR of 50%, DCR of 83.3%, median PFS of 7.58 months, and a surgical conversion rate of 47.2%, with 16 patients achieving R0 resection and a pCR in 31.3%, though grade ≥ 3 TRAEs occurred in 52.6% of participants (104). In the second-line setting, an open-label phase II study (NCT05148195) of envafolimab (300 mg every 3 weeks) in combination with the anti-VEGF antibody suvemcitug established a recommended suvemcitug dose of 2 mg/kg every 3 weeks and, among 20 previously treated patients, demonstrated an ORR of 10%, DCR of 65%, median PFS of 4.3 months, and median OS of 10.7 months, with grade ≥ 3 TRAEs in 40% of patients (105). Finally, a case report of a 57-year-old male receiving envafolimab for HCC described acute onset of insulin-deficient diabetes mellitus and diabetic ketoacidosis without detectable diabetes-related autoantibodies, underscoring the importance of vigilant monitoring for severe irAEs and the need for biomarker development to predict both efficacy and toxicity in ICI therapies (106).

A phase II, non-randomized, open-label study (NCT05148195) investigated the combination of envafolimab, suvemcitug, and FOLFIRI in patients with microsatellite stability (MSS)/proficient mismatch repair (pMMR) CRC in the second-line setting. The recommended dose of suvemcitug was established at 2 mg/kg biweekly in combination with envafolimab and FOLFIRI. Among 20 enrolled patients, the ORR was 25.0% and the DCR reached 90%, with a median PFS of 5.6 months. No DLTs were observed, and the most common grade ≥3 TRAEs included neutropenia, leukopenia, and hypertension, indicating a manageable safety profile (107).

### 4.4 Enhancing envafolimab efficacy through epigenetic modulation

Epigenetic agents, such as azacitidine, a DNA methyltransferase inhibitor, function by demethylating DNA and reprogramming the gene expression profile of tumor cells. This process can lead to the re-expression of tumor-associated antigens and immune-modulatory genes that were previously silenced through hypermethylation. Reactivating the expression of these antigens increases the tumor cells’ visibility to the immune system, thereby enhancing their recognition and targeting by T cells. Furthermore, epigenetic agents can elevate the expression of MHC molecules on tumor cells, improving their ability to present antigens to T cells. With enhanced antigen presentation, anti-PD-L1 agents can more effectively block the PD-L1/PD-1 interaction, thereby preventing T cell exhaustion and sustaining T cell activity. Tumors often develop resistance to immunotherapy through mechanisms such as epigenetic changes that downregulate antigen presentation. By reversing these changes, epigenetic agents may enhance the efficacy of anti-PD-L1 agents. This topic has been extensively reviewed in recent literature and will not be discussed in detail here (108–115).

NSCLC patients who are resistant to anti-PD-1 therapy present a significant therapeutic challenge. Recent studies have suggested that combining histone deacetylase inhibitors (HDACs), such as chidamide, with anti-PD-L1 inhibitors may yield synergistic anti-tumor effects. A clinical study was conducted to evaluate the therapeutic potential of this combination in advanced patients who had previously failed anti-PD-1 treatment. While the overall response was modest, the findings indicated that patients with high HDAC2 expression, negative PD-L1 status, and low blood tumor mutational burden (bTMB) may derive greater benefit from this combination. This suggests a potential epigenetic mechanism contributing to the observed responses. These results highlight the need for further investigation into biomarkers that can predict response to this combination therapy, as well as the necessity for larger clinical trials to confirm these preliminary findings and refine patient selection for precision treatment strategies in advanced NSCLC (116).

### 4.5 Other combination approaches involving envafolimab

The ChiCTR2100054796 clinical trial explored a novel combinatorial approach aimed at overcoming resistance to immune checkpoint blockade therapy in NSCLC, specifically in patients who had previously failed anti-PD-1 treatment. The strategy combined β-glucan, a biologically active polysaccharide known for its immune-modulating properties, with envafolimab and endostar (a recombinant human endostatin as antiangiogenic agent).

23 NSCLC patients were given β-glucan (500 mg twice daily for 21 days), envafolimab (300 mg on day 1), and endostar (210 mg via 72-hour continuous infusion) administered every 3 weeks. The reported ORR was 21.7%, while DCR and median PFS were 73.9% and 4.3 months overall respectively, with a significant difference based on PD-L1 status: PD-L1 positive: 6.3 months and PD-L1 negative: 2.3 months. This trial offers early but compelling evidence that β-glucan can modulate the TME to improve responses to ICIs in NSCLC. The inclusion of endostar, targeting tumor vasculature, may further contribute to improved drug delivery and immune infiltration. Notably, the observed increase in efficacy among PD-L1-positive patients aligns with existing knowledge on checkpoint blockade, though the combination’s utility in PD-L1-negative disease, despite lower median PFS, still suggests some benefit. From a translational perspective, the proteomic analysis is particularly valuable. Among the proteins elevated in non-responders, caspase-8 (CASP-8), arginase-1 (ARG1), matrix metalloproteinase 12 (MMP12), CD28, and C-X-C motif chemokine ligand 5 (CXCL5) were identified. CASP-8, a key apoptotic regulator, may reflect disrupted cell death pathways that enable tumor cells to evade immune-mediated apoptosis. Elevated ARG1 levels, indicative of immunosuppressive MDSCs and TAMs, suggest active suppression of T-cell responses via arginine depletion. MMP12, involved in extracellular matrix degradation and tissue remodeling, is known to facilitate tumor progression and may hinder immune cell infiltration. Interestingly, increased CD28, despite being a co-stimulatory molecule, may signal T-cell dysfunction or exhaustion in the context of chronic antigen exposure. CXCL5, a chemokine that recruits neutrophils and promotes inflammatory cascades, has been implicated in creating an immunosuppressive TME. Conversely, favorable responses to therapy were associated with elevated levels of CD40 ligand (CD40-L) and epidermal growth factor (EGF). CD40-L is a critical co-stimulatory molecule that enhances APC function and supports T-cell activation, highlighting a mechanism of effective immune engagement. While EGF is typically associated with oncogenic signaling, its presence in responders may reflect a more immunologically active or targetable tumor phenotype in this specific therapeutic context. These findings underscore the complex interplay of immune modulatory pathways in the TME and provide preliminary evidence that the balance of immunosuppressive versus immunostimulatory signals influences treatment outcomes. Furthermore, the data support the hypothesis that β-glucan contributes to immune reprogramming, potentially overcoming resistance by altering the composition and function of immune components within the TME. It not only supports the biological rationale for the combination but also lays the groundwork for developing predictive biomarkers to refine patient selection in future studies (117).

A growing body of evidence highlights the therapeutic versatility of envafolimab beyond conventional systemic immunotherapy, particularly when integrated with locoregional strategies in HCC. In this context, the development of biodegradable microsphere-based delivery systems represents a significant innovation. Specifically, envafolimab-loaded hyaluronic acid–gelatin microspheres (envafolimab-MS) enable sustained intra-arterial drug release following TACE, effectively overcoming key limitations associated with standard intravenous or subcutaneous administration, such as rapid systemic clearance and suboptimal intratumoral drug concentration. Mechanistically, this platform directly counteracts TACE-induced expansion of PD-L1^+^ MDSCs, a critical driver of post-treatment immune escape, while simultaneously reprogramming the TME toward an immunostimulatory state. Preclinical models demonstrate that envafolimab-MS enhances CD4^+^ and CD8^+^ T-cell activation, promotes M1 macrophage polarization, increases innate lymphocyte infiltration, and reduces immunosuppressive cell populations, collectively resulting in superior tumor growth inhibition compared with free antibody administration. Importantly, localized delivery prolongs intratumoral drug retention and minimizes systemic exposure, thereby improving both efficacy and safety profiles. Furthermore, the embolization capability of the microspheres provides an additional antitumor mechanism via vascular occlusion, reinforcing the synergistic interaction between immunotherapy and TACE. Collectively, these findings position envafolimab-based microsphere delivery as a promising, clinically translatable strategy that not only enhances drug bioavailability but also fundamentally reshapes tumor–immune dynamics to reduce recurrence and improve therapeutic outcomes in HCC (70).

A retrospective single-center study evaluated the efficacy of anlotinib combined with envafolimab in 15 patients with unresectable or metastatic liposarcoma. The cohort included predominantly dedifferentiated histology (73.3%), with an ORR of 6.7% and DCR of 73.3%. The median PFS was 14.2 months, and grade 3–4 TRAEs occurred in 42.2% of patients, with liver function abnormalities, hypertension, and fatigue being the most common (118).

A phase II, open-label, single-arm study (ENLEN-OC-001) evaluated a novel “home-based” therapeutic regimen combining the subcutaneously administered PD-L1 inhibitor envafolimab with the oral antiangiogenic agent lenvatinib and the chemotherapeutic drug etoposide. This triplet approach demonstrated encouraging antitumor activity, with an objective response rate (ORR) of 44.4% and a DCR of 83.3%, alongside a median progression-free survival (PFS) of 10.2 months and OS of 21.3 months. These outcomes compare favorably with previously reported results for other immune checkpoint inhibitor (ICI)-based combinations in similar patient populations, suggesting a potential additive or synergistic benefit derived from simultaneous targeting of immune checkpoints, tumor angiogenesis, and DNA damage pathways.

Importantly, the safety profile of the regimen was manageable, with no treatment-related deaths or unexpected toxicities observed. The most common grade 3–4 adverse events were hematological, particularly leukopenia and thrombocytopenia, largely attributable to etoposide and generally reversible with dose adjustments. IrAEs were relatively limited, with hypothyroidism being the most frequently reported. Notably, the fully outpatient, minimally invasive administration—subcutaneous injection combined with oral agents—did not adversely affect patients’ quality of life or psychological well-being, highlighting a significant advantage over traditional infusion-based regimens.

Mechanistically, this combination strategy is supported by a strong biological rationale. PD-L1 blockade restores T-cell–mediated antitumor immunity, while lenvatinib-mediated inhibition of VEGF/VEGFR signaling promotes vascular normalization and enhances immune cell infiltration within the TME. Concurrently, etoposide-induced DNA damage may increase tumor immunogenicity and further stimulate immune responses. Together, these effects may synergistically disrupt tumor immune evasion and improve therapeutic outcomes. Although exploratory biomarker analyses did not demonstrate a clear association between PD-L1 expression and response, observations such as favorable outcomes in clear cell carcinoma and in a patient with HRD suggest that tumor biology may influence responsiveness and warrants further investigation.

Despite these promising findings, limitations including the small sample size, single-arm design, and relatively short follow-up period must be acknowledged. Nevertheless, this study provides preliminary evidence supporting the feasibility, efficacy, and tolerability of a convenient, home-based combination immunotherapy regimen. Further randomized, large-scale trials are needed to validate these results, optimize patient selection, and clarify the role of predictive biomarkers in guiding treatment with envafolimab-based combinations in ovarian cancer (119).

This phase II single-center prospective study by Tian et al. explores a postoperative triplet regimen combining the subcutaneous PD-L1 inhibitor envafolimab with capecitabine and lenvatinib in patients with resected biliary tract cancer (BTC) at high risk of recurrence. The study addresses a clinically important gap, as recurrence after R0 resection remains frequent despite standard capecitabine-based adjuvant therapy. The reported median disease-free survival (DFS) of 15.63 months and 1-year DFS rate of 68.3%, along with a 1-year OS of 91.4%, suggest encouraging early disease control in a biologically aggressive cohort enriched for lymph node metastasis, perineural invasion, and lymphovascular invasion. Importantly, the safety profile appears manageable, with no treatment-related deaths and grade ≥3 adverse events consistent with expected toxicities of chemotherapy and anti-angiogenic therapy. However, the interpretation of efficacy is constrained by the single-arm design, small sample size, and lack of a contemporary control group, making it difficult to distinguish treatment effect from patient selection and favorable baseline characteristics. The heterogeneity of BTC subtypes and the relatively short follow-up further limit definitive conclusions regarding long-term survival benefit. Nonetheless, the exploratory biomarker analysis highlighting cancer antigen 19–9 as a potential predictor of early recurrence adds translational value and aligns with prior evidence supporting its prognostic relevance. Overall, this study provides hypothesis-generating clinical evidence that combining PD-L1 blockade with anti-angiogenic therapy and chemotherapy may enhance postoperative disease control in high-risk BTC, but validation in adequately powered randomized controlled trials with standardized comparator arms is essential before clinical adoption (120).

The summary of clinical data on envafolimab were provided in **Table 1**.

**Table 1:** Summary of the key details from the studies involving envafolimab safety and efficacy in cancer patients.

NCT identifier /reported title	Phase	Patients	Dosage	Tumor type	Key findings	Adverse effects	Clinical Outcomes	PMID
NCT02827968	Phase I	28	0.01–10 mg/kg weekly following a modified 3 + 3 design. In a dose-exploration phase, patients received subcutaneous envafolimab 300 mg once every 4 weeks	Advanced solid tumors	A direct relationship between dosage and plasma concentration. Median time to peak concentration: 4–7 days. Terminal half-life of 14 days after dose 1 in cycle 1 and 23 days at steady state.	No DLTs or injection site reactions.	PR in 3 patients.	33973293
NCT03248843	Phase I	35	In the dose-escalation phase, 10 patients received subcutaneous envafolimab once a week at 1.0 mg/kg, 2.5 mg/kg, and 5.0 mg/kg. In the dose-expansion phase, 16 patients were treated at 2.5 or 5.0 mg/kg twice a week in part 1, and 9 patients received envafolimab 300 mg once every four weeks in part 2.	Advanced solid tumors	High tolerability with no unknown adverse effects. A dose-dependent increase in AUC and C_max.	Grade ≥ 3 adverse events: adrenal insufficiency, cerebral infarction, immune-mediated enterocolitis.	ORR of 11.4%; DCR of 34.3%	35932387
NCT03667170	Phase II	103	150 mg weekly in a 28-day treatment cycle	Advanced or metastatic malignant dMMR/MSI-H solid tumors	Demonstrated effectiveness and safety in patients with advanced dMMR/MSI-H tumors.	Acceptable safety profile	ORR of 42.7%; DCR of 66.0%, PFS of 11.1 months	34154614
Efficacy and safety of combining short-course neoadjuvant chemoradiotherapy with envafolimab in locally advanced rectal cancer patients with microsatellite stability: A phase II PRECAM experimental study	Phase II	34	Short-course radiation delivered 25Gy to the primary tumor and high-risk areas. Patients received two cycles of CAPEOX chemotherapy and envafolimab immunotherapy after radiotherapy. After two weeks of neoadjuvant therapy, TME surgery was conducted. The three-dimensional conformal or intensity-modulated radiation treatment followed clinical practice, irradiating within seven days with a dose split of 5Gy per fraction and a total dosage of 25Gy per 5 fractions. The CAPEOX chemotherapy regimen included oxaliplatin (130 mg/m2, day 1) and capecitabine (1000 mg/m2, taken orally on days 1-14). Envafolimab was subcutaneously injected at 150 mg weekly for six weeks with CAPEOX.	Locally advanced rectal cancer (LARC)	Favorable efficacy and safety in LARC	Common adverse events included tenesmus (78.1%), diarrhea (62.5%), and leukocyte decrease (40.6%). Two Grade 3 adverse events were noted, one related to liver function abnormality and the other to a decrease in platelet count	pCR rate of 62.5%; pathologic response rate of 75% in MSS tumors.	39093871
Chidamide plus envafolimab as subsequent treatment in advanced non-small cell lung cancer patients resistant to anti-PD-1 therapy: A multicohort, open-label, phase II trial with biomarker analysis	Phase II	Total 34: preliminary study (N = 3), formal study (N = 31)	Preliminary study: chidamide 20 mg/twice a week + envolimab 400 mg every 4 weeks. Formal study: chidamide 30 mg/twice a week + envolimab 400 mg every 4 weeks.	Advanced non‐small cell lung cancer (NSCLC)	Modest overall response. High HDAC2 expression, negative PD-L1 status, low bTMB might predict better outcomes.	Anemia, decreased platelet count, hypokalemia, decreased neutrophil count and hypoalbuminemia	ORR of 6.7%; DCR of 50%; median PFS of 3.5 months	38597130
ChiCTR2100054796	Phase I/II	23 NSCLC patients (prior anti-PD 1 treatment failure)	β-glucan 500 mg orally, twice daily on days 1–21; Envafolimab 300 mg intravenously on day 1, every 3 weeks (once every 21 days); Endostar 210 mg continuously through intravenous infusion over 72 h repeated every 3 weeks	Advanced non‐small cell lung cancer (NSCLC)	proteomic markers (resistance: CASP-8, ARG1, MMP12, CD28, CXCL5; response: CD40-L, EGF)	TRAEs in 52.2% (hypothyroidism 26.1%, fatigue 26.1%); grade 3 in 8.7%; no deaths	ORR of 21.7%; DCR of 73.9%; median PFS of 4.3 months (PD-L1^+^ 6.3 months vs PD-L1^-^ 2.3 months)	39271997
NCT05213221	Phase II	36 HCC patients	Transcatheter arterial chemoembolization (TACE), envafolimab (300 mg, subcutaneous injection every 3 weeks) and lenvatinib (12 mg (≥60 kg) or 8 mg (<60 kg) orally, once daily	Unresectable hepatocellular carcinoma (HCC)	Envafolimab combined with lenvatinib and TACE produced encouraging survival rates and conversion efficacy, accompanied by an acceptable safety profile.	TRAEs of any grade was 97.4%. Grade ≥ 3 were recorded in 52.6% of patients. No fatalities associated with the treatment were reported.	ORR of 50%; DCR of 83.3%	39384742
ChiCTR2200055407	Phase II	30 HCC patients	Envafolimab (150 mg, once every week, subcutaneous) in combination with lenvatinib (12 mg for patients weighing over 60 kg, 8 mg for patients weighing under 60 kg).	Inoperable hepatocellular carcinoma (HCC)	Envafolimab combined with lenvatinib demonstrated favorable anti-cancer efficacy and a tolerable safety profile for the first-line treatment of patients with HCC.	Twenty-three patients (76.7%) encountered at least one TRAE, with increased aspartate aminotransferase (AST) being the most prevalent at 23.3%.	Median OS of 18.5 months; median PFS of 9.4 months; ORR of 40%; DCR of 80%	39690337
NCT05148195	Phase II	20 HCC patients	Envafolimab (300 mg every 3 weeks) in combination with the anti-VEGF antibody suvemcitug established a recommended suvemcitug dose of 2 mg/kg every 3 weeks	Hepatocellular carcinoma (HCC)	The combination of envafolimab and suvemcitug demonstrated an acceptable safety profile and encouraging antitumor efficacy in HCC patients who had unsuccessful later-line treatment.	40% of patients experienced TRAEs of grade ≥ 3, with proteinuria being the most common.	ORR of 10%, DCR of 65%, median PFS of 4.3 months, and median OS of 10.7 months	39883265
NCT05148195	Phase II	20	Envafolimab (300 mg every 3 weeks) + suvemcitug (2 mg/kg every 3 weeks) + FOLFIRI	MSS/pMMR CRC	Manageable safety profile; preliminary anti-tumor efficacy in second-line MSS/pMMR CRC	Most common grade ≥3 TRAEs: decreased neutrophil and white blood cell counts, hypertension	ORR of 25%; DCR 90%; DoR 4.1 months; median PFS of 5.6 months; OS not reached	39836256
Anlotinib combined with envafolimab in unresectable or metastatic liposarcoma	Not specified (retrospective study)	15	Anlotinib + envafolimab (dosing not specified)	Unresectable or metastatic liposarcoma	Promising efficacy in advanced liposarcoma; disease control in majority	TRAEs in 7 patients; grade 3–4 in 3 patients (42.2%); most common: liver function abnormalities, hypertension, fatigue	ORR of 6.7%; DCR of 73.3%; median PFS of 14.2 months	39868378

ARG1, arginase 1; AUC, area under the concentration-time curve; bTMB, blood tumor mutational burden; CAPEOX, capecitabine plus oxaliplatin; C_max, maximum plasma concentration; CASP-8, caspase 8; CD28, cluster of differentiation 28; CD40L, CD40 ligand (also known as Tumor Necrosis Factor Superfamily Member 5, TNFSF5); CRC, colorectal cancer; CXCL5, C-X-C Motif Chemokine Ligand 5; dMMR, deficient mismatch repair; DCR, disease control rate; DLTs - Dose-Limiting Toxicities; DoR, duration of response; EGF, epidermal growth factor; Gy, Gray (unit of radiation dose); HDAC2, histone deacetylase 2; HCC, hepatocellular carcinoma; LARC, locally advanced rectal cancer; mg/kg, milligrams per kilogram; MMP, matrix metalloproteinase; MMR, mismatch repair; MSS, microsatellite stability; MSI-H, microsatellite instability-high; NCT, national clinical trial; NSCLC, non-small cell lung cancer; ORR, objective response rate; OS, overall survival; PD-1, programmed cell death protein 1; PD-L1, programmed cell death ligand 1; PFS, progression-free survival; pCR, pathologic complete response; pMMR, proficient MMR; PR, partial response; PRECAM, Phase II PRECAM experimental study; TACE, transcatheter arterial chemoembolization; TME, tumor microenvironment; TRAEs, treatment-related adverse events; VEGF, vascular endothelial growth factor.

In an interesting study by Dong et al. (121), nineteen patients with advanced gastric adenocarcinoma were treated with envafolimab either as monotherapy or in combination with other therapies. Notably, four patients with low PD-L1 expression achieved an ORR of 75%, with a DCR of 100% (121).

Collectively, the clinical data indicate that envafolimab exhibits a favorable safety profile, pharmacokinetic stability, and clinically meaningful antitumor activity across multiple tumor types. However, several limitations should be considered when interpreting these findings. Early-phase studies consistently demonstrated predictable dose exposure, prolonged half-life, and manageable immune-related toxicities, supporting the feasibility of subcutaneous administration and fixed dosing regimens. This pharmacological profile represents an important practical advantage over intravenously administered PD-1/PD-L1 antibodies, particularly by improving treatment accessibility and reducing healthcare burden. Nevertheless, the majority of available studies are single-arm, involve relatively small patient cohorts, and frequently lack direct comparative control groups, limiting definitive conclusions regarding the superiority of envafolimab over established PD-1/PD-L1 inhibitors. The strongest clinical evidence currently supports its activity in biomarker-selected populations, particularly dMMR/MSI-H tumors, where high TMB and increased neoantigen generation provide a biologically favorable environment for immune checkpoint blockade. In contrast, responses in less immunogenic tumors, including MSS CRC and previously treated solid tumors, remain modest and highlight the challenge of primary and acquired resistance to PD-L1 inhibition. Combination studies with chemotherapy, radiotherapy, antiangiogenic agents, and epigenetic modulators suggest that envafolimab may have broader therapeutic potential by remodeling the TME and enhancing immune activation. However, these approaches remain exploratory and require validation in randomized clinical trials. Importantly, the identification of predictive biomarkers remains a major unmet need, as *PD-L1* expression alone appears insufficient to reliably select patients likely to benefit from envafolimab. Future investigations integrating genomic, transcriptomic, proteomic, and immune profiling will be essential to define optimal patient populations, clarify mechanisms of resistance, and determine whether the unique structural and pharmacological characteristics of envafolimab translate into improved clinical outcomes compared with conventional checkpoint inhibitors.

## 5 Future and ongoing clinical trials

Promising results from completed clinical trials have spurred the development of additional studies. Currently, ten phase I, seventy phase II, and four phase III clinical trials involving envafolimab are registered on ClinicalTrials.gov (https://clinicaltrials.gov/search?cond=Cancer&intr=Envafolimab, accessed on 24.06.2026).

Envafolimab is currently being tested in phase II clinical trials in combination with conventional chemotherapies, including docetaxel (NCT05910034; NSCLC), capecitabine (NCT06202014; locally advanced pancreatic cancer), capecitabine and oxaliplatin (XELOX) (NCT05335460 and NCT06014372; advanced colon cancer), gemcitabine and cisplatin (GEMOX) (NCT06013943; advanced gallbladder cancer), nab-paclitaxel combined with cisplatin or carboplatin (NCT06218004 and NCT05828381; head and neck squamous cell carcinoma and esophageal cancer, respectively), and XELOX in combination with short-course radiotherapy (NCT05858567; MSS locally advanced ultra-low rectal adenocarcinoma).

Radiotherapy targets and eradicates localized tumors and stimulates a systemic immune response against the tumor, often referred to as the abscopal effect. Radioimmunotherapy regimens frequently incorporate stereotactic body radiation therapy (SBRT), a technique that allows precise delivery of high doses of radiation using image-guided and intensity-modulated methods. Similarly, chemotherapy-induced ICD results in the release or exposure of DAMPs such as CRT, ATP, and HMGB1. This process enhances antigen presentation, promotes the depletion of Tregs, increases T-cell infiltration, facilitates macrophage repolarization, and upregulates PD-L1 expression on cancer cells, thereby increasing the efficacy of anti-PD-L1 antibodies (122).

In the context of curative cancer treatment, the integration of chemotherapy and radiotherapy with ICIs has gained considerable attention, particularly for its potential application in metastatic disease. Various sequencing strategies, induction, concurrent, and consolidation, offer distinct potential benefits. The induction approach, where ICIs precede chemotherapy or radiotherapy, may enhance immune priming and facilitate earlier systemic control of the disease. Concurrent administration, where chemotherapy or radiotherapy is paired with ICIs, capitalizes on the synergy between these modalities, potentially exploiting chemotherapy/radiotherapy-induced tumor antigen release and associated immunologic shifts. A consolidation strategy, in which ICIs follow definitive chemotherapy or radiotherapy, aims to sustain immune activation and provide long-term surveillance against residual disease. Despite concerns about increased toxicity with concurrent treatment, early clinical data do not indicate a significant rise in adverse events. Current evidence suggests that the therapeutic efficacy of combining chemotherapy or radiotherapy with ICIs is likely optimized when these modalities are administered in close temporal proximity. However, the lack of definitive evidence favoring one sequencing strategy over another highlights the need for further investigation. Tumor type and stage may significantly influence the optimal approach, and ongoing clinical trials are crucial to better define these factors. Continued support for these efforts is essential to refine treatment paradigms and improve patient outcomes (123).

When optimizing radioimmunotherapy, selecting appropriate radiotherapy regimens is crucial for enhancing therapeutic efficacy while minimizing adverse effects. Traditional radiotherapy, typically developed without considering its impact on the immune system, can inadvertently diminish the effectiveness of combined immunotherapy due to immune system suppression, including lymphopenia. Hypofractionated radiotherapy, which involves delivering radiation in larger doses over fewer sessions, has shown promise in augmenting antitumor immunity when combined with ICIs. This approach appears to be more effective than conventional fractionation, as evidenced by studies demonstrating enhanced tumor control with regimens like 8Gy × 2 compared to 2Gy × 10. The choice of radiotherapy fractionation and dosing is, therefore, a critical factor in optimizing the synergy between radiotherapy and immunotherapy. The timing of immunotherapy administration relative to radiotherapy is another key consideration. Evidence suggests that concurrent administration of radiotherapy and ICIs may lead to better long-term tumor control compared to sequential treatment, potentially due to the immediate immune-priming effects of radiotherapy. However, this is not universally supported across all studies, indicating that the optimal sequence may vary based on factors such as the type of cancer, the specific ICI used, and individual patient characteristics. Moreover, even sequential administration can result in prolonged PFS, underscoring the complexity of determining the ideal timing for combined therapies. To further refine radioimmunotherapy, reducing the radiation field to spare critical immune-related structures is essential. Techniques like intensity-modulated radiotherapy (IMRT) allow for more precise targeting of tumors while minimizing exposure to lymph nodes, bone marrow, and circulating blood cells, which are vital for maintaining an effective immune response. Protecting these structures not only preserves the immune system’s ability to mount a robust response but also enhances the potential for systemic, abscopal effects, where localized radiotherapy leads to regression of non-irradiated tumors. Advancements in radiotherapy technologies, such as proton therapy, heavy ion radiotherapy (HIRT), and FLASH radiotherapy, offer further opportunities to reduce collateral damage to the immune system. Proton therapy and HIRT provide more precise energy delivery to tumors, reducing exposure to surrounding healthy tissue and potentially enhancing immune activation. FLASH radiotherapy, with its ultra-high dose rate, minimizes treatment duration and the exposure of circulating immune cells, potentially reducing normal tissue toxicity while maintaining tumor control. These innovative approaches hold significant promise for improving the outcomes of radioimmunotherapy, although more research is needed to fully understand their benefits in combination with immunotherapy, as discussed by other authors (124–130).

TME is highly intricate and characterized by a dynamic interaction between immunosuppressive and immunostimulatory components. This necessitates the collaboration of many established efficacious treatments within the context of vascular normalization. The systemic administration of medications faces difficulty in reaching the intended cancer cells due to a compromised, impaired, and malfunctioning vascular system. In contrast, intralesional administration of drugs is ineffective in achieving diffusion. The use of antiangiogenic multikinase inhibitors is crucial to normalize the vascularization. Reversal of the blockage in the lymphatic channels due to tumor response does not indicate restoration of lymphatic function. To enable the flow of new antigens to the lymph nodes and activate the immune response, a TGF-β, VEGFR1–3, FGFR1–4 or platelet-derived growth factor receptor alpha (PDGFRα) blocker may be necessary. The death of cancer cells and the accumulation of their remaining constituents cause edema and raise interstitial pressure. This necessitates the prompt initiation of professional phagocytosis to eliminate the dead cells. Pharmaceuticals that promote the process of professional phagocytosis, along with immunostimulant autophagy, enhance the successful infiltration of TILs and the completion of an immune response cycle. Furthermore, cancer cells possess an extraordinary mutational capacity, enabling them to overcome initially effective treatments. This genetic plasticity creates a continuous spectrum of potential mechanisms of resistance, underscoring the need for ongoing innovation in therapeutic strategies. The use of pulsed radiation or SBRT to induce the production of neoantigens, tailored to the specific mutations of a tumor, enhances the body’s immune response by boosting the activity of memory immune cells. This approach also aligns with the need for supplementary dosing to augment the effectiveness of immunotherapy. Therefore, strategically applying SBRT to specific lesions before each round of antiangiogenic treatment, administered either in a cyclical, regular dose, or low continuous manner, in combination with immunotherapy, holds significant promise as an effective treatment strategy, as previously discussed by Swamy (131–133).

In line with the available evidence, envafolimab is being evaluated in several phase II clinical trials in combination with various therapeutic agents for the treatment of solid tumors. These trials include combinations with lenvatinib for endometrial cancer (NCT05112991), epithelial ovarian cancer (NCT05422183), HCC (NCT05213221, NCT05582109), and other solid tumors (NCT05024214, NCT05422183). Additionally, envafolimab is being tested in combination with trifluridine/tipiracil and bevacizumab for metastatic CRC (NCT06060704), fruquintinib for advanced or unresectable locally advanced bone and soft tissue sarcoma (NCT05941325), and endostar either alone or in conjunction with SBRT for advanced gastrointestinal tumors (NCT05203276, NCT06301828). Other ongoing studies include combinations with chemotherapy in advanced pancreatic cancer (NCT05298020) and locally advanced nasopharyngeal carcinoma (NCT06055816), donafenib for recurrent HCC (NCT06498622), surufatinib for advanced soft tissue sarcoma (NCT05722977), and endostatin in conjunction with chemotherapy NSCLC and squamous NSCLC (NCT05360979, NCT05529355, NCT05243355). Additionally, envafolimab is being tested in combination with S-1 (an oral fluoropyrimidine derivative that combines tegafur, gimeracil, and oteracil), oxaliplatin, short-course radiotherapy, and endostatin for locally advanced gastric cancer (NCT05387681).

LM-101 is an investigational humanized monoclonal antibody developed by LaNova Medicines that targets signal regulatory protein alpha (SIRPα), a key immune checkpoint receptor expressed on myeloid cells such as macrophages. By binding to SIRPα, LM-101 disrupts the SIRPα–CD47 interaction, which typically delivers a “don’t eat me” signal to immune cells. This blockade enhances the phagocytic activity of macrophages against tumor cells, potentially restoring innate immune surveillance and promoting anti-tumor responses. LM-101 has shown promising anti-tumor effects in animal models, which led to its advancement into clinical trials for patients with advanced cancers. Further research is ongoing, exploring LM-101 both as a monotherapy and in combination with other anti-tumor agents, including envafolimab (NCT05615974; advanced malignant tumors, phase I/II; the study is currently suspended).

By inhibiting multiple immune checkpoints, combination therapy can modulate the immune response more comprehensively. For instance, while anti-PD-L1 agents primarily affect T cells within the TME, cytotoxic T-lymphocyte-associated protein 4 (CTLA-4) inhibitors can enhance T cell priming and activation in lymph nodes. Tumors often use multiple redundant pathways to suppress immune responses. Blocking several pathways at once can prevent the tumor from compensating by upregulating alternative checkpoints. Envafolimab is currently investigated in combination with CTLA-4 inhibitors including YH001, with or without doxorubicin for advanced or metastatic sarcoma (NCT05448820; phase I/II study) or ipilimumab in patients with undifferentiated pleomorphic sarcoma or myxofibrosarcoma (NCT04480502).

Alphamab has developed JSKN033, a subcutaneous injectable medication composed of bispecific antibodies targeting human epidermal growth factor receptor 2 (HER2), consisting of JSKN003 and envafolimab. JSKN003 consists of a cleavable linker, an inhibitor of topoisomerase I, and bispecific antibodies that target two non-overlapping epitopes of the HER2 extracellular domain. JSKN033–101 is an open-label, multicenter phase I/II study encompassing dose expansion and dose escalation studies. In the phase I dose escalation stage, the objective is to evaluate the safety, tolerability, PK, and preliminary efficacy of JSKN033 in patients who have advanced or metastatic HER2-expressing solid tumors. This will be accomplished by determining the maximum tolerated dose (MTD) and/or the phase II recommended dose (RP2D). In the phase II dose expansion stage, the efficacy and safety of JSKN033 will be evaluated in HER2-expressing gastrointestinal tumors at the RP2D dose (NCT06226766, advanced or metastatic solid malignant tumors, phase I/II study).

**Figure 3:**
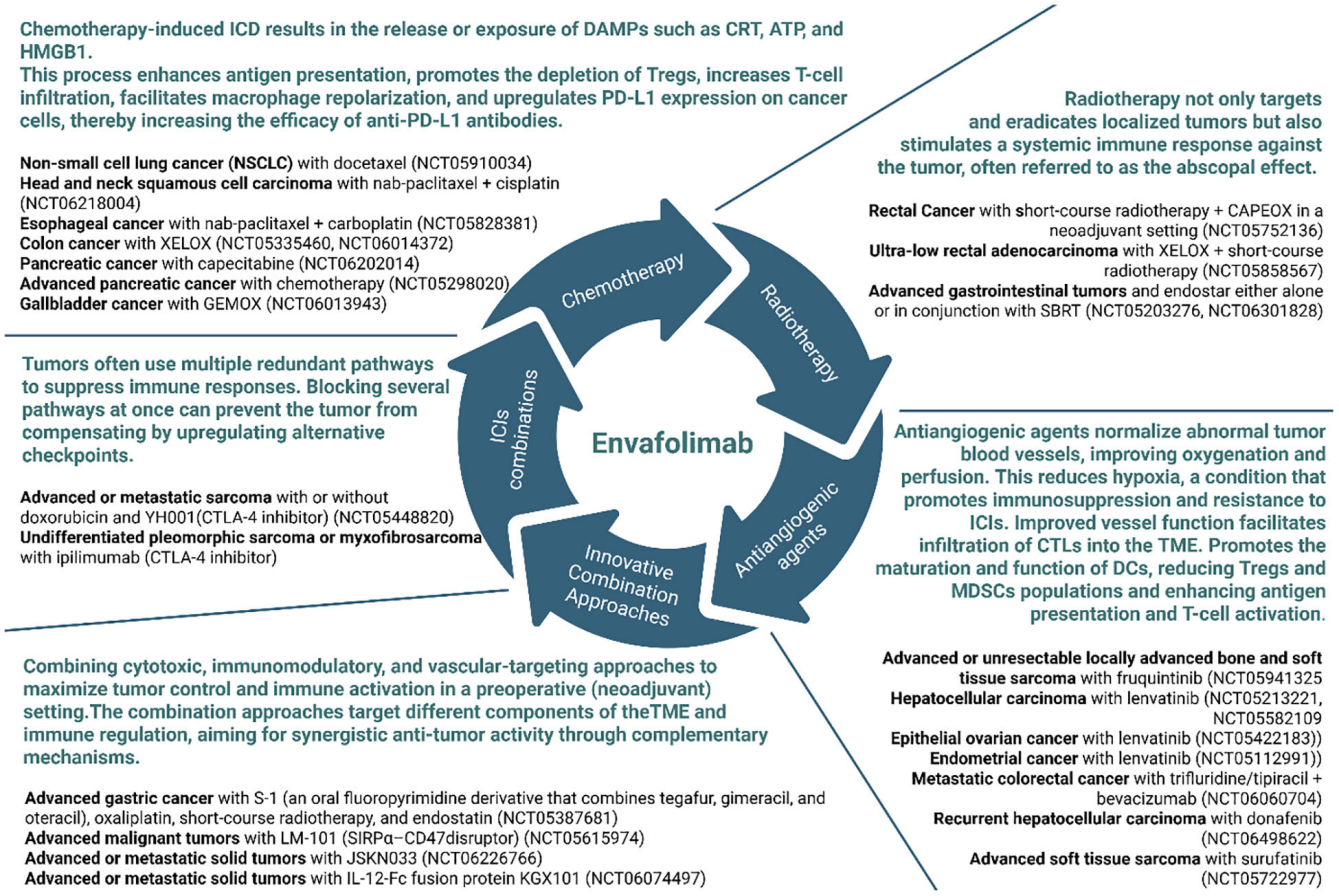
Ongoing and future clinical trials investigating envafolimab with chemotherapy, radiotherapy, antiangiogenic agents, other immune checkpoint inhibitors (ICIs), as well as, novel combinational approaches. For full list of abbreviations, see Glossary section. Created in BioRender. Kciuk, M. (2026) https://BioRender.com/g1l3ba0.

The IL-12-Fc fusion protein (KGX101) is an engineered therapeutic agent designed to harness the immune system’s natural ability to target and destroy cancer cells. This fusion protein consists of the human IL-12 linked to an Fc domain, which is masked by protein domains to prevent premature activation. When administered, the IL-12 in KGX101 remains pharmacologically inactive due to the binding of these masking proteins. The activation of IL-12 is specifically controlled by tumor-specific MMPs present within the TME. Upon cleavage by these enzymes, IL-12 is released and becomes active within the local tumor environment. Once activated, IL-12 stimulates the immune system by inducing IFN-γ production and activating critical immune cells, such as CD8+ T cells, CD4+ T cells, and NK cells. These immune cells initiate cytolytic responses that specifically target and destroy tumor cells, inhibiting their proliferation. The Fc domain in KGX101 also serves to prolong the protein’s half-life, thus increasing its duration of activity and exposure within the tumor, potentially enhancing its therapeutic effectiveness. An open-label, two-part, multicenter, multi-regional phase I trial (NCT06074497) is underway to explore the safety, tolerability, and pharmacokinetic features of KGX101. This trial will also investigate its efficacy in combination with envafolimab in patients with advanced or metastatic solid tumors. Selected future clinical trials including envafolimab along with chemotherapy, radiotherapy, antiangiogenic agents, other ICIs, as well as, novel combinational approaches were summarized in **Figure 3**.

Interestingly, there are three phase III clinical trials registered on clinicaltrials.gov (https://clinicaltrials.gov/search?cond=cancer&intr=envafolimab%20&aggFilters=phase:3 accessed on 24.06.2026) that investigate envafolimab. The purpose of the first study (NCT05752136) is to explore and evaluate a new preoperative neoadjuvant treatment model for LARC. This new model combines short-course radiotherapy with immunotherapy (including envafolimab) and CAPEOX chemotherapy. The study aims to determine the feasibility, efficacy, and adverse effects of this treatment approach. Additionally, it seeks to identify characteristics of patients who benefit from the treatment through multi-omics sequencing analysis, to develop an efficacy prediction model for precise, individualized treatment.

The purpose of the second study (NCT06123754) is to evaluate the efficacy and safety of envafolimab combined with platinum-based doublet chemotherapy compared to placebo combined with platinum-based doublet chemotherapy as neoadjuvant/adjuvant therapy in patients with resectable stage IIIA and IIIB (N2) (NSCLC). The study aims to determine how well this treatment approach works by assessing the major pathological response (MPR) and event-free survival (EFS) as primary endpoints. Additionally, the study will monitor the safety of the treatment and the outcomes related to tumor progression and patient survival.

Third study (NCT06959693), is designed to evaluate the efficacy and safety of envafolimab in combination with cetuximab-β and mFOLFOX6 in patients with MSS, *RAS*/serine/threonine-protein kinase (*BRAF*) wild-type metastatic CRC. The study includes patients with unresectable CRC who have not received prior systemic therapy for metastatic or recurrent disease. In Phase II, approximately 186 patients will be randomized (1:1) to receive either the investigational combination therapy or standard treatment. In Phase III, about 404 patients will be similarly randomized. Following a screening period of up to 28 days, patients will receive treatment in 2-week cycles for up to 2 years, followed by a safety and survival follow-up every 12 weeks.

The ongoing clinical development program reflects a broad effort to expand the therapeutic applications of envafolimab beyond biomarker-selected populations by evaluating rational combination strategies targeting complementary mechanisms of tumor immune evasion. The diversity of ongoing phase II and III studies, encompassing chemotherapy, radiotherapy, antiangiogenic agents, dual immune checkpoint blockade, and novel immunomodulatory therapies, underscores the versatility of envafolimab as a potential backbone for combination immunotherapy. Nevertheless, the current clinical landscape remains fragmented, with many studies investigating distinct tumor types, treatment settings, and combination regimens, which may complicate cross-study comparisons and delay the identification of optimal therapeutic strategies. Furthermore, many of the investigated combinations are supported primarily by strong biological rationale and preclinical evidence, whereas their clinical efficacy and safety remain to be demonstrated. Whether these approaches will overcome primary or acquired resistance to PD-1/PD-L1 blockade, broaden the benefit to immunologically “cold” tumors, or improve long-term survival without unacceptable toxicity remains uncertain. Future studies should also strengthen the integration of predictive biomarkers with the unique pharmacological characteristics of envafolimab. In addition to established biomarkers such as *PD-L1* expression, MSI-H/dMMR status, TMB, and features of the TME, the identification of biomarkers specifically associated with response to envafolimab may facilitate more precise patient selection. Moreover, the distinctive single-domain antibody structure of envafolimab provides opportunities for further optimization through antibody engineering to improve affinity, stability, and tumor penetration, as well as through advanced drug-delivery technologies aimed at extending residence time, prolonging systemic half-life, and maximizing therapeutic exposure while preserving the advantages of subcutaneous administration. The results of ongoing randomized clinical trials will therefore be essential not only to establish the clinical value of individual combination strategies but also to identify biomarkers that enable more precise patient selection and guide personalized treatment approaches.

## 6 Variability of response and other enhancement strategies

### 6.1 Influence of DDR pathway

Proficient DNA mismatch repair and DNA polymerase proofreading maintain genomic stability. Mutations in DNA polymerase epsilon, catalytic subunit (*POLE*) and DNA polymerase delta 1, catalytic subunit (*POLD1*) genes generate an ultramutated phenotype with high TMB and increased neoantigen formation, resulting in enhanced immune infiltration and improved responses to immune checkpoint inhibitors, even in MSS tumors. Accordingly, POLE/POLD1 mutations are being investigated as predictive biomarkers for immunotherapy, although their low prevalence limits broad clinical applicability (134–137).

Heat shock proteins (HSPs) are a diverse category of highly conserved chaperone proteins that exhibit a wide array of enzymatic functions. HSPs facilitate the folding, stabilization, and membrane translocation of nascent and stress-denatured proteins, a capacity that becomes hijacked in cancer cells to buffer the proteotoxic burden imposed by oncogenic mutations and rapid proliferation. Accordingly, tumors often exhibit marked upregulation of HSPs, which in turn preserve the conformational integrity of mutant oncoproteins and support malignant phenotypes. Under conditions of oxidative stress, nutrient deprivation, or therapy-induced cytotoxicity, HSP-peptide complexes are released, either passively via necrotic lysis or actively through secretory pathways (including extracellular milieu HSPs, membrane-associated HSPs, and extracellular vesicle–associated HSPs), into the TME These extracellular HSPs exert dichotomous immunomodulatory effects that hinge on their mode of release: necrosis-associated HSP–tumor antigen complexes are endocytosed by APCs, enabling efficient cross-presentation on MHC class I and II molecules, activation of cytotoxic CD8^+^ T lymphocytes, helper CD4^+^ T cells, and subsequent humoral responses. In contrast, exocytosed or vesicular HSPs frequently promote immune tolerance by driving T-reg cell expansion, MDSC activation, and the secretion of anti-inflammatory cytokines (e.g., IL-10, IL-6), thereby fostering an immunosuppressive milieu conducive to tumor progression. Notably, specific HSP family members exhibit distinct extracellular activities: HSP60 engages TLR-4 to expand CD4^+^CD25^+^forkhead box P3^+^(Foxp3^+^) Tregs and induce IL-10 production, while HSP27 skews monocyte differentiation toward angiogenic, immunotolerant macrophages, and EV-HSP72 potentiates myeloid suppressor functions. Conversely, membrane-bound HSP70 can recruit and activate NK cells, underscoring the context-dependent balance between HSP-driven immune activation and evasion. Collectively, these insights delineate a dualistic role for HSPs in tumor biology, both as enablers of oncogenic proteostasis and as key arbiters of the immunological synapse and highlight their potential as targets or vehicles in novel immuno‐oncological strategies. This topic was recently reviewed by Boliukh et al. (138), Das et al. (139) and Mazurakova et al. (140).

Additionally, their involvement in the DNA damage response (DDR) pathway is beginning to emerge (141). For example, HSP90 was shown to determine the choice of BER pathway through interaction with X-ray repair cross-complementing protein 1 (XRCC1) (142), while stabilizing MSH2 protein involved in MMR repair (143). HSP90 was also shown to stabilize the DNA-dependent protein kinase, catalytic subunit (DNA-PKcs) and promote efficient NHEJ (144), while at the same time promoting DNA repair protein RAD51 homolog 1 (RAD51) (145) and MRN complex formation during HR (146). Furthermore, HSP90 was shown to modulate the response to DNA damage through its impact on ataxia telangiectasia and Rad3 related (ATR) (147) and Ataxia-telangiectasia mutated (ATM) (148) serine/threonine kinases.

Consistent with this concept, the HSP90 inhibitor TAS-116 (pimitespib) combined with nivolumab demonstrated encouraging activity in MSS CRC, supporting further investigation of DDR-modulating strategies alongside checkpoint inhibition, including envafolimab (149).

### 6.2 High/low immunoscore

Numerous research has been conducted to determine the predictive importance of TILs in CRC, as well as the therapeutic implications of the tumor immune microenvironment changes. For example, the clinical prognosis of human CRC can be predicted based on the kind, abundance, and spatial distribution of immune cells inside the TME (150). The so-called “immunoscore” quantifies the concentration of lymphocytes, namely CD3+ and CD8+ T cells, in both the central region and the invasive edges of the tumor. This scoring system ranges from a low immune cell density (immunoscore 0) to a high density (immunoscore 4). The essential aspect of this process is that tumors with a higher level of immunogenicity have a greater ability to recruit T-cells as well as other components of TME involved in anti-cancer immune response, including DCs, macrophages, and NK cells. High immunoscore is associated with a longer PFS, whereas patients with a low immunoscore and modest tumor invasion are more prone to disease relapse (151). These observations suggest that baseline immune infiltration may help identify patients most likely to benefit from envafolimab.

### 6.3 Effect of gut microbiome

Growing evidence indicates that the gut microbiome influences responses to immune checkpoint inhibitors through modulation of systemic and local antitumor immunity. Specific bacterial communities have been associated with improved responses to PD-1/PD-L1 blockade. Specifically, these patients exhibited elevated amounts of *Enterococcus faecium*, *Bifidobacterium longum*, and *Collinsella aerofaciens* in their colons. Furthermore, antibiotic consumption was shown to affect the microbiome composition leading to reduced response to ICIs or the development of toxic side effects. This topic was previously discussed in excellent reviews and is beyond the scope of this work (152–155). According to these findings, the ICONIC phase II trial will be conducted to investigate the impact of neoadjuvant chemotherapy consisting of oxaliplatin and capecitabine in conjunction with immunotherapy and adjuvant therapy on the regression of locally advanced MSS colon cancer. The use of *Clostridium butyricum* as an adjuvant will be used to investigate the possibility of further enhancing the significant response rate of neoadjuvant therapy (NCT05914389).

### 6.4 Other factors

**Figure 4:**
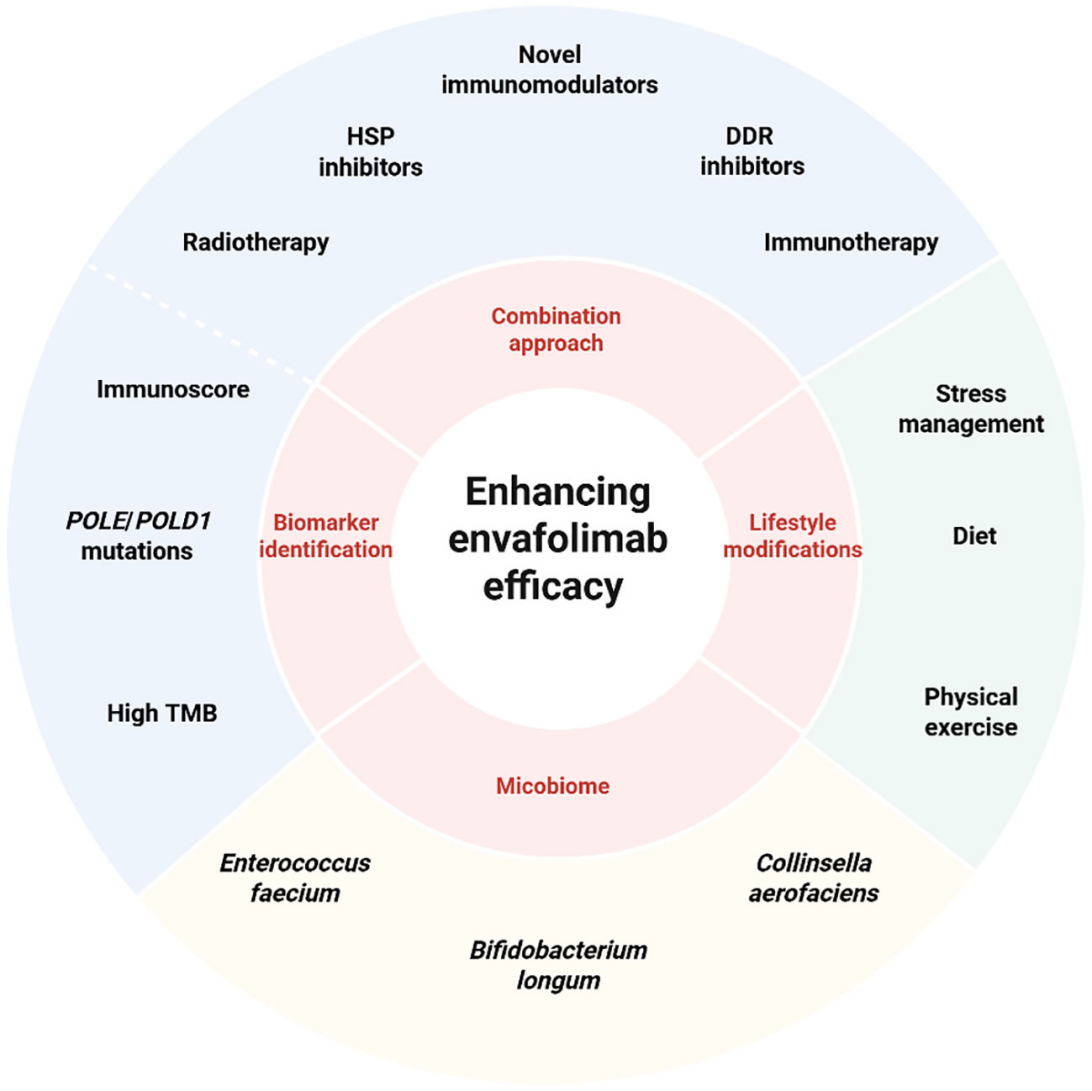
Summary factors relevant to the design of future envafolimab-based studies based on previous investigations of anti-programmed death receptor (PD-1)/programmed death-ligand 1 (PD-L1) antibodies. Created in BioRender. Kciuk, M. (2026) https://BioRender.com/8kj5ehu.

Additional host-related factors, including age, sex, race, lifestyle, metabolic disorders, physical activity, stress, and diet, may also affect immunotherapy outcomes and should be considered when designing future clinical studies of envafolimab (153, 154). **Figure 4** outlines critical factors to be considered in the rational design of future clinical investigations of envafolimab.

## 7 Conclusions and prospects

Envafolimab represents a significant advancement in the field of immuno-oncology, leveraging its innovative design as a humanized single-domain PD-L1 antibody fused with a human IgG1 Fc fragment. Its unique subcutaneous administration route not only enhances patient comfort and compliance but also introduces the possibility of at-home administration, addressing logistical and accessibility challenges.

Clinical trials involving envafolimab demonstrated promising results that triggered the investigations of the drug in combination with chemotherapy, radiotherapy, and other immunomodulatory agents. These combinations aim to exploit synergies such as enhanced tumor antigen release, improved immune activation, and complementary therapeutic effects. Envafolimab’s role in cutting-edge strategies, such as radioimmunotherapy and integration with novel immunomodulatory modalities, highlights its adaptability and broad-spectrum potential across various tumor types and treatment stages.

The ongoing development of combination therapies with agents like CTLA-4 inhibitors, angiogenesis modulators, and emerging biologics further extends the drug’s utility. For example, combinations involving envafolimab and innovative agents such as JSKN033, KGX101, and LM-101 illustrate the commitment to addressing resistant or refractory cancers.

Looking ahead, the continued exploration of envafolimab in phase II and III clinical trials is pivotal to defining its role in both early- and advanced-stage cancers. Studies focusing on patient stratification, efficacy prediction through multi-omics, and personalization of treatment regimens are expected to refine their application and maximize outcomes. For example, routine detection of mutations in the catalytic subunit of *POLE*, which are associated with high TMB and the development of a “hot” TME rich in cytotoxic T lymphocytes (CTLs) should be performed as they have been shown to correlate with improved responses to ICIs. Furthermore, advancements in delivery mechanisms and the incorporation of novel immunotherapy techniques may position envafolimab as a cornerstone in integrated cancer treatment strategies. With its unique pharmacokinetic properties and expanding clinical applications, envafolimab is poised to contribute significantly to the evolution of precision oncology.

In addition to tumor-intrinsic factors, modulation of the gut microbiome has emerged as a pivotal determinant of immunotherapy success. Specific microbial taxa, including *Enterococcus faecium* and *Bifidobacterium longum*, have been linked to favorable responses to PD-1 inhibitors. Incorporating adjuvants like *Clostridium butyricum* into therapeutic regimens may synergistically enhance immune responses in patients treated with envafolimab, as evidenced by ongoing trials in CRC.

Furthermore, behavioral and lifestyle interventions, such as stress management, physical exercise, and dietary modifications, have demonstrated the potential to modulate immune function and improve TME. These approaches, in conjunction with pharmacological agents like envafolimab, could offer a comprehensive strategy to overcome resistance mechanisms and improve therapeutic efficacy.

Although the influence of these host-related factors on immunotherapy efficacy has been investigated in the context of other anti-PD-1/PD-L1 antibodies, their predictive or prognostic value in patients treated with envafolimab remains largely unexplored. Current evidence regarding the impact of age, sex, lifestyle-related factors, metabolic status, physical activity, psychological stress, and dietary patterns on immune checkpoint inhibitor outcomes is primarily derived from studies involving agents such as nivolumab, pembrolizumab, atezolizumab, and other approved checkpoint inhibitors. Whether these associations can be extrapolated to envafolimab, particularly given its distinct subcutaneous administration route and pharmacokinetic profile, remains to be determined. Prospective studies incorporating comprehensive host-factor profiling alongside clinical and molecular biomarkers will be required to clarify their relevance in predicting response, resistance, and toxicity in patients receiving envafolimab.

## Glossary

AEs: adverse events
APCs: antigen-presenting cells
ARG1: arginase-1
ATM: ataxia-telangiectasia mutated
ATP: adenosine triphosphate
ATR: ataxia telangiectasia and Rad3 related
AUC: area under the concentration-time curve
BER: base excision repair
BRAF: serine/threonine-protein kinase B-Raf
bTMB: blood tumor mutational burden
CAFs: cancer-associated fibroblasts
CAPEOX: capecitabine and oxaliplatin (chemotherapy regimen)
CASP-8: caspase-8
CAR-T: chimeric antigen receptor T-cell
C_max: maximum plasma concentration
CCL3: C-C motif chemokine ligand 3
CD25+: cluster of differentiation 25 positive
CD40-L: CD 40 ligand
CD274: cluster of differentiation 274 (another name for PD-L1)
CD279: cluster of differentiation 279 (another name for PD-1)
CDR3: complementarity-determining region 3
CR: clinical complete response
CRC: colorectal cancer
CRT: calreticulin
ctDNA: circulating tumor DNA
CTLs: cytotoxic T lymphocytes
CTLA-4: cytotoxic T-lymphocyte-associated protein 4
CXCL5: C-X-C motif chemokine ligand 5
CXCL8: C-X-C motif chemokine ligand 8
CXCL10: C-X-C motif chemokine ligand 10
DAMPs: damage-associated molecular patterns
DCs: dendritic cells
DCR: disease control rate
DDR: DNA damage response
DDX20: DEAD-box helicase 20
dMMR: deficient mismatch repair
DLTs: dose-limiting toxicities
DNA-PKcs: DNA-dependent protein kinase, catalytic subunit
EDMs: exonuclease domain mutations
EFS: event-free survival
EGF: epidermal growth factor
EGFR: epidermal growth factor receptor
Fc: fragment crystllizable
FDA: Food and Drug Administration
FGFR: fibroblast growth factor receptor
FOXP3: forkhead box P3
GEMOX: Gemcitabine and Cisplatin (chemotherapy regimen)
GZMA/B: granzyme A/B
Gy: Gray (unit of radiation dose)
HCAbs: heavy-chain antibodies
HCC: hepatocellular carcinoma
HDAC: histone deacetylase
HER2: human epidermal growth factor receptor 2
HGF: hepatocyte growth factor
HIFs: hypoxia-inducible factors
HMGB1: high mobility group box 1
HPV: human papillomavirus
HR: homologous recombination
HRD: homologous recombination deficiency
HSP90: heat shock protein 90
HSPs: heat shock proteins
ICD: immunogenic cell death
IFNs: interferons
IFN-γ: interferon-gamma
ICI: immune checkpoint inhibitor
IL: interleukin
Immunoscore: immune scoring system
IMRT: intensity-modulated radiotherapy
IRF: interferon regulatory factor
KGX101: IL-12-Fc fusion protein
KIT: tyrosine kinase kit
KRAS: Kirsten rat sarcoma viral oncogene
LARC: locally advanced rectal cancer
MDSCs: myeloid-derived suppressor cells
MHC: major histocompatibility complex
MMPs: matrix metalloproteinases
MMR: mismatch repair
MPR: major pathological response
MSH2: MutS homolog 2
MSI-H: high microsatellite instability
MSS: microsatellite stable
MTD: maximum tolerated dose
NCT: National Clinical Trial Number
NF-κB: nuclear factor kappa-B
NHEJ: non-homologous end joining
NK: natural killer cells
NSCLC: non-small cell lung cancer
ORR: overall response rate
OS: overall survival
P2RX7: P2X purinoceptor 7
PARP: poly (ADP-ribose) polymerase
PBMCs: peripheral blood mononuclear cells
PD-1: programmed death-1
PD-L1: programmed death ligand-1
PDGFR-β: platelet-derived growth factor receptor β
PFNs: perforins
PFS: progression-free survival
PK: pharmacokinetics
pMMR: proficient mismatch repair
POLD1: DNA polymerase delta 1, catalytic subunit
POLE: DNA polymerase epsilon, catalytic subunit
PR: partial response
RAD51: DNA repair and recombination protein RAD51
RAF: rapidly accelerated fibrosarcoma
RET: rearranged during transfection
RP2D: recommended phase 2 dose
SBRT: stereotactic body radiation therapy
SD: stable disease
siRNA: small interfering RNA
SIRPα: signal regulatory protein alpha
SMIs: small-molecule inhibitors
TACE: transcatheter arterial chemoembolization
TAMs: tumor-associated macrophages
TCR: T-cell receptor
TEAEs: treatment-emergent adverse events
TGF-β: transforming growth factor-beta
TIE2: TEK tyrosine kinase
TILs: tumor-infiltrating lymphocytes
TLR-4: toll-like receptor 4
TME: tumor microenvironment
TNF-α: tumor necrosis factor-alpha
TRAEs: treatment-related adverse events
T-regs: regulatory T cells
VEGF: vascular endothelial growth factor
VEGFR1-3: vascular endothelial growth factor receptors 1–3
XRCC1: X-ray repair cross-complementing protein 1
